# Low electric current in a bioelectrochemical system facilitates ethanol production from CO using CO-enriched mixed culture

**DOI:** 10.3389/fmicb.2024.1438758

**Published:** 2024-08-29

**Authors:** Chaeho Im, Minsoo Kim, Jung Rae Kim, Kaspar Valgepea, Oskar Modin, Yvonne Nygård, Carl Johan Franzén

**Affiliations:** ^1^Division of Industrial Biotechnology, Department of Life Sciences, Chalmers University of Technology, Göteborg, Sweden; ^2^School of Chemical Engineering, Pusan National University, Busan, Republic of Korea; ^3^Institute of Bioengineering, University of Tartu, Tartu, Estonia; ^4^Division of Water Environment Technology, Department of Architecture and Civil Engineering, Chalmers University of Technology, Göteborg, Sweden; ^5^VTT Technical Research Centre of Finland, Espoo, Finland

**Keywords:** bioelectrochemical system, microbial electrosynthesis, bioethanol, gas fermentation, carbon monoxide, acetogen, *Clostridium autoethanogenum*

## Abstract

Fossil resources must be replaced by renewable resources in production systems to mitigate green-house gas emissions and combat climate change. Electro-fermentation utilizes a bioelectrochemical system (BES) to valorize industrial and municipal waste. Current electro-fermentation research is mainly focused on microbial electrosynthesis using CO_2_ for producing commodity chemicals and replacing petroleum-based infrastructures. However, slow production rates and low titers of metabolites during CO_2_-based microbial electrosynthesis impede its implementation to the real application in the near future. On the other hand, CO is a highly reactive gas and an abundant feedstock discharged from fossil fuel-based industry. Here, we investigated CO and CO_2_ electro-fermentation, using a CO-enriched culture. Fresh cow fecal waste was enriched under an atmosphere of 50% CO and 20% CO_2_ in N_2_ using serial cultivation. The CO-enriched culture was dominated by *Clostridium autoethanogenum* (≥89%) and showed electro-activity in a BES reactor with CO_2_ sparging. When 50% CO was included in the 20% CO_2_ gas with 10 mA applied current, acetate and ethanol were produced up to 12.9 ± 2.7 mM and 2.7 ± 1.1 mM, respectively. The coulombic efficiency was estimated to 148% ± 8% without an electron mediator. At 25 mA, the culture showed faster initial growth and acetate production but no ethanol production, and only at 86% ± 4% coulombic efficiency. The maximum optical density (OD) of 10 mA and 25 mA reactors were 0.29 ± 0.07 and 0.41 ± 0.03, respectively, whereas it was 0.77 ± 0.19 without electric current. These results show that CO electro-fermentation at low current can be an alternative way of valorizing industrial waste gas using a bioelectrochemical system.

## 1 Introduction

Electro-fermentation is a promising technology to valorize residual carbons using renewable energy (Jiang et al., 2019). Both gaseous carbon (e.g., CO_2_) and organic wastes (e.g., municipal and agricultural waste) can be used as substrates for electro-fermentation. Gasification of such waste results in so-called syngas, containing CO, CO_2_ and H_2_. Depending on the oxidation state of the carbon being used as a substrate in a bioelectrochemical system (BES), various products can be produced, either by anodic or cathodic electro-fermentation (Chandrasekhar et al., 2021). Gaseous carbon substrate for cathodic electro-fermentation usually refers to CO_2_ to contribute to the Sustainable Development Goal 13, Climate Action, aiming at net zero greenhouse gas emissions by 2050 in accordance with the Paris Agreement (United Nations Environment Programme, 2015). Electro-fermentation requires lower power consumption than traditional catalytic electrosynthesis, resulting in carbon-negative chemical production with the help of renewable energy (Jiang et al., 2019; Chu et al., 2020). CO_2_ is a favored substrate for cathodic electro-fermentation, because it leads to valorization of residual CO_2_ that would otherwise both be wasted and contribute to climate change. The main products of CO_2_ electro-fermentation are methane and/or acetate, depending on operational conditions such as the composition of the medium and of the mixed culture inoculum (Marshall et al., 2012; Samanides et al., 2020). Recent studies have also reported production of ethanol and butanol, as well as short- and medium-chain fatty acids, from gaseous carbon in BESs using mixed cultures already enriched in BES reactors (Vassilev et al., 2018; Romans-Casas et al., 2024). Therefore, cathodic electro-fermentation is a viable option for capturing and valorizing CO_2_. Nevertheless, low titers and selectivity of products, along with a diverse product spectrum causing increased cost in product recovery, challenges the implementation of CO_2_-based electro-fermentation in the near future (Claassens et al., 2019; Prevoteau et al., 2020).

Gas fermentation employs acetogens as biocatalysts, such as *Clostridium ljungdahlii* and *Clostridium autoethanogenum* (Tanner et al., 1993; Abrini et al., 1994). These acetogens utilize the Wood-Ljungdahl pathway to fix one-carbon molecules, specifically CO and CO_2_, converting them into acetyl-CoA (Fackler et al., 2021). From this pivotal central metabolite, a variety of valuable chemicals can be synthesized (Liew et al., 2016). Understanding and optimizing CO conversion is important as CO is a major component of syngas and industrial flue gas. Moreover, the standard redox potential of the CO_2_/CO half-cell (−520 mV) is more negative than that of the 2H^+^/H_2_ half-cell (−420 mV). This means that CO conversion has more potential to produce reduced metabolites than the conversion of CO_2_ as a sole carbon source with the help of H_2_ oxidation (Chu et al., 2020). Some of the possible reactions that lead to ethanol formation from CO_2_ and CO are listed in Eqs. 1–4.


(1) $$2{\mathrm{CO}}_{2}+6H_{2}\to C_{2}H_{5}\mathrm{OH}+3H_{2}O\;\left(\Delta G=-97.4\mathrm{kJ}/\mathrm{mol}\right)$$

(2) $$2\mathrm{CO}+4H_{2}\to C_{2}H_{5}\mathrm{OH}+H_{2}O\;\left(\Delta G^{{}^{\circ}}=-137.6\mathrm{kJ}/\mathrm{mol}\right)$$

(3) $$3\mathrm{CO}+3H_{2}\to C_{2}H_{5}\mathrm{OH}+{\mathrm{CO}}_{2}\;\left(\Delta G^{{}^{\circ}}=-157.7\mathrm{kJ}/\mathrm{mol}\right)$$

(4) $$6\mathrm{CO}+3H_{2}O\to C_{2}H_{5}\mathrm{OH}+4{\mathrm{CO}}_{2}\;\left(\Delta G=-218.0\mathrm{kJ}/\mathrm{mol}\right)$$

Focusing on CO conversion can significantly increase the overall efficiency of value-added chemical production (Im et al., 2018). In addition to electro-fermentation, industrial waste gases and gasified solid organic waste can be utilized using gas fermentation without the need of supplying electrical current to the cultures. Gas fermentation is already technologically mature enough to be commercially viable. That this technology can replace at least part of the petrochemical industry is illustrated by LanzaTech, which is one of the leading companies commercializing ethanol from syngas fermentation and actively broadening the product spectrum (Mihalcea et al., 2023). Moreover, the efficient conversion of CO into useful products via acetogens not only has industrial relevance but also bears considerable environmental importance (Badr and Probert, 1995). One limitation of CO as the sole or main substrate is that CO has low solubility in water (27.6 mg/L at 25°C), which limits the performance of syngas fermentation (Phillips et al., 2017). Moreover, when H_2_ supply is insufficient, two-thirds of the carbons from CO are lost as CO_2_ (Eq. 4) (Valgepea et al., 2018). When fermenting syngas three-fold more H_2_ than CO_2_ are required to convert CO_2_ into ethanol (Eq. 1), and not many types of syngas contain such high percentage of hydrogen (Ciliberti et al., 2020). In this case, a BES can be an option to supply extra reducing equivalents for fixing CO_2_ and to improve carbon yields of products (Im et al., 2018; Song et al., 2020, 2021).

CO conversion in a BES was first studied using the electron mediator neutral red (Im et al., 2018). The authors suggested that electrons supplied from the electrode to the bacterial cells via an electron mediator could increase product yields from CO conversion by lowering CO_2_ production in the BES (Im et al., 2018). Although several operational parameters were modulated to increase the performance of the microbial electrosynthetic CO conversion, low titers of short-chain fatty acids dominated the product spectrum, with low selectivity (Song et al., 2020, 2021). In order to evaluate the use of industrial waste gas as an alternative to pure CO_2_, Chu et al., tested different CO/CO_2_ ratios in a BES using a mixed culture and fixed current without an electron mediator (Chu et al., 2020). Even though an optimal CO/CO_2_ ratio was found for better CO consumption and caproate selectivity during CO electro-fermentation compared to other conditions, both the coulombic efficiency and the selectivity towards caproate stayed low (<100 and 15.4%, respectively) (Chu et al., 2020). Moreover, the complexity of the mixed culture makes it difficult to identify paths for further improvement. Pre-enriching the mixed culture under conditions suitable for the study may provide important information for CO electro-fermentation achieving higher CE and higher titers of metabolites produced with high selectivity (Patil et al., 2015).

Im et al., tested different cathode potentials ranging from −0.6 to −1.2 (vs 3 M Ag/AgCl,) for CO_2_ microbial electrosynthesis using *Clostridium ljungdahlii* (Im et al., 2022). In the study, cathode potentials at −0.8 V and −1.0 V (vs 3 M Ag/AgCl), corresponding to currents about 10 mA and 25 mA, respectively, resulted in CEs close to 100% (Im et al., 2022). When a cathode potential of −1.2 V (vs 3 M Ag/AgCl) was applied, the performance (in terms of CE, productivity, and cell growth) was instead decreased (Im et al., 2022). These results suggest that higher current driven by higher cathodic potential does not lead to better performance.

In this study, cow fecal waste was enriched under CO before being used as an inoculum in electro-fermentation experiments. Different fixed currents (10 mA and 25 mA) and different gaseous substrates (CO/CO_2_ mix and CO_2_ only) were tested to investigate the performance of the inoculum in a BES. When 10 mA was applied to the BES reactor, improved cell viability and ethanol conversion was observed. The results of this study indicate important parameters to consider when a CO-containing gas mixture is used in a BES.

## 2 Materials and methods

### 2.1 The source of inoculum and medium composition

In order to maintain near-anaerobic conditions, a 50 mL Falcon-tube was fully filled with fresh cow fecal waste from a cow farm located in Yangsan-si, South Korea. The sample tube used for inoculation was moved into a Coy anaerobic chamber within an hour after sample collection.

The medium used in serum flasks contained: K_2_HPO_4_, 0.35 g/L; KH_2_PO_4_, 0.23 g/L; NH_4_Cl, 1.0 g/L; KCl, 0.1 g/L; MgSO_4_⋅7H_2_O, 0.2 g/L; NaCl, 0.8 g/L; CaCl_2_⋅2H_2_O, 0.02 g/L; sodium acetate, 0.25 g/L; yeast extract, 1.0 g/L; L-Cysteine⋅HCl, 0.6 g/L; 1 mL/L of 1 g/L resazurin, and 10 mL/L of mineral solution and vitamin solution, respectively. The composition of the mineral and vitamin solutions were the same as in DSMZ medium 879 (Im et al., 2022). The medium was prepared anaerobically, and the pH of the medium was set to pH 5.0. Prior to inoculation, 5 mM of sodium 2-bromoethanesulfonate was added to the medium to inhibit methanogenesis during the enrichment process.

### 2.2 Inoculum enrichment of cow fecal waste under CO

The enrichment process was adapted from Grimalt-Alemany et al. (2018). Briefly, 5 g of fresh cow fecal waste was inoculated into 50 mL medium in a 250 mL serum flask. The headspace was evacuated and filled with 2 bars of ultra-high purity [N_2_:CO:CO_2_] = [30:50:20] gas (99.999%, impurities <5 ppm, GD Gas, Korea). The headspace gas was replaced when the overpressure was lower than 0.5 bar. Liquid samples were taken every 24 h to measure pH and metabolite concentrations. Whenever the pH stopped decreasing and no more changes in metabolite production were detected, 10% of the culture was transferred to another serum bottle containing 50 mL of fresh medium. After ten rounds of such serial cultivations, the enriched culture was used as inoculum for BES experiments. All cultivations were done in a temperature-controlled incubator at 37°C.

The primary source of CO for use in electro-fermentation is synthesis gas (syngas). The CO content in syngas varies from 13 to 63% (Ciliberti et al., 2020; Girod et al., 2020). In this study, a moderately high CO concentration (50%) was chosen to test the potential inhibitory effect of CO on extracellular electron transfer. H_2_ was omitted to see the effect of electric current applied without interference from H_2_ consumption.

### 2.3 Bioelectrochemical system operation

H-type BES reactors (Adams and Chittenden Scientific Glass Coop, Berkeley, CA, United States) were used for bioelectrochemical system operation. An Aquivion^®^ E98-09S cation exchange membrane (Solvary Specialty Polymers, United States) was used to separate the cathode and anode chambers. Each chamber contained 250 mL of the culture medium, but without resazurin and with yeast extract added only in the cathode chamber. A graphite felt (2.5 cm × 4 cm; GF065, Fuel cell store, United States) was used as the cathode and a platinized titanium wire as the anode (MAGNETO special anodes, The Netherlands). Ultra-high purity [N_2_:CO:CO_2_] = [30:50:20] gas was sparged continuously through a stainless needle (Merck, United States) connected to a needle valve at a rate of 10 mL/min. Constant currents were applied to the BES reactors using a multi-channel potentiostat (WMPG1000K, WonA Tech, Korea). The BES reactors were operated in a temperature-controlled incubator at 37°C. Each cathode chamber of the BES reactors was stirred using a magnetic bar at 50 rpm throughout the experiment. The enriched culture was inoculated to the cathode chamber to an OD of 0.05 to initiate the experiments. Prior to testing CO electro-fermentation, CO_2_ electro-fermentation was done to test electro-activity of the CO-enriched culture. CO_2_ electro-fermentation was done in duplicates, and CO electro-fermentation in triplicates.

### 2.4 Analysis

Using a syringe, a 1 mL liquid sample was taken every 24 h from the serum bottle during the enrichment and from the cathode and the anode chambers during BES reactor operation. Bacterial cell growth was estimated by measuring the optical density (OD) using a spectrophotometer (WPA S1200+, Biochrom, Cambridge, United Kingdom) at a wavelength of 600 nm in 1 cm light path.

Samples for metabolite quantification were stored at −20°C until analysis. After thawing, the samples were centrifuged and filtered through a syringe filter (0.22 μm, PTFE-H, Korea). The filtered samples were analyzed using an HPLC system equipped with a refractive index detector (at 40°C) and a UV detector (at 210 nm) (Jasco, Japan). The ROA-Organic acid H+ (8%) column (Rezex, Torrance, CA, United States) was installed in the oven and the oven temperature was maintained at 60°C, using 5 mM H_2_SO_4_ as the mobile phase at 0.6 mL/min.

The headspace of cultures was analyzed by gas chromatography (GC, 6500GC, YL Instrument, Korea) equipped with a Porapak N column (10 ft. × 1/8 in × 2.1 mm) and Mol sieve 13X (3 ft. × 1/8 in × 2.1 mm). Gaseous carbon molecules (CH_4_ and CO_2_) were detected using a flame ionization detector (FID), and other gas components, such as hydrogen and nitrogen, were detected using a thermal conductivity detector (TCD). Argon was used as the carrier gas. 100 μL of the gas samples were introduced directly into the injector using a pressure-lock syringe (100 μL, Hamilton, United States).

The diversity and taxonomic composition of the bacterial community of the CO-enriched culture was characterized by next-generation sequencing (NGS, Macrogen, Korea). Both the V1–V2 (27F-Eub338 primer set) and V3–V4 (Bakt_341F-805R primer set) regions of the 16S rRNA gene were analyzed to increase the resolving power for identifying bacterial taxa. Briefly, Maxwell^®^ 16 Tissue DNA Purification kit (AS1030, Promega, United States) was used to purify DNA from the sample. Afterwards, Herculase II Fusion DNA Polymerase Nextera XT Index V2 Kit was used for the library preparation and Illumina Miseq platform for the sequencing. FLASH (1.2.11) was used for merging assembly of the paired end reads from the original DNA fragments (Magoc and Salzberg, 2011). Raw read filtering and trimming, error-free reads picking, and operational taxonomic units (OTU) clustering at different distance cutoffs (0.03) were performed using CD-HIT-OTU and rDnaTools (Li et al., 2012). QIIME was used to assign taxonomy with NCBI_16S_20230103 (BLAST) (Sequences with ≥97% similarity at species level).

### 2.5 Coulombic efficiency

The CE is the ratio of coulombs recovered in products to coulombs input to the system (Eq. 5), calculated as


(5) $$\mathrm{CE}\;\left(\%\right)=\frac{F\sum_{i}b_{i}n_{i}}{\int_{0}^{t}Idt}\times 100\%,$$

where *F* corresponds to Faraday’s constant (96,485 C/mol), *b_i_* is the number of electrons required in the synthesis of the product *i* (mol e^−^/mol product), *n_i_* is the amount of product *i* (mol), and *i* is the electrical current (A) through the BES circuit at time *t*. The CE of each experiment was calculated based on the CO_2_ reduction into acetate and ethanol (Eqs. 6, 7) (Im et al., 2022). The products detected by HPLC, both from the cathode and the anode chambers, were considered for CE calculations as product diffusion through a proton exchange membrane was reported (Im et al., 2022).


(6) $$2{\mathrm{CO}}_{2}+8e^{-}+8H^{+}\to {\mathrm{CH}}_{3}\mathrm{COOH}+2H_{2}O$$

(7) $$2{\mathrm{CO}}_{2}+12e^{-}+12H^{+}\to {\mathrm{CH}}_{3}{\mathrm{CH}}_{2}\mathrm{OH}+3H_{2}O$$

### 2.6 Cyclic voltammetry

CV measurements were done at the end of the CO_2_ electro-fermentation experiment and abiotic experiment to compare bacterial activities. The CV was measured from −1.1 V to 0.2 V vs. an Ag/AgCl (3 M KCl) reference electrode at a scan rate of 1 mV/s. Cathode potential mentioned throughout this manuscript is compared to Ag/AgCl (3 M NaCl) reference electrode.

### 2.7 Statistical analysis

Statistical analysis was performed using R software, function *t*.test(), for two-tailed *t*-test. Statistical significance was established at *p*-value <0.05.

## 3 Results

### 3.1 Bacterial community after enrichment in CO

Four weeks after inoculation of the initial cow fecal waste in serum flasks pressurized to 2 bar with a gas mixture of 30% N_2_, 50% CO and 20% CO_2_, CO consumption and acetate production was detected. After the initial batch, serial sub-culturing was performed after every 7–9 days. The early cultures produced acetate, ethanol, butyrate, and butanol (data not shown). After ten serial batch cultivations, the enriched microbial culture produced only acetate and ethanol.

The enriched bacterial community was analyzed using 16S rRNA sequencing. The majority of the bacterial community (≥89%) was found to consist of *Clostridium autoethanogenum* according to the sequencing service (Table 1). The results were consistent between the V1–V2 and the V3–V4 primer pairs for the 16S rRNA gene. However, *Clostridium autoethanogenum* cannot be distinguished from the closely related *Clostridium ljungdahlii* on the 16S RNA level, as discussed below. The second most frequent bacterial species of the enriched community was identified to be the lactic acid bacterium *Lacticaseobacillus paracasei* (7.5% using primers V1–V2) or *Lacticaseobacillus chiayiensis* (4% with primers V3–V4). *Lacticaseibacillus* spp., *Enterococcus* spp., and the rest of the bacterial community, are non-autotrophic bacteria, which were present in the population in relatively small amounts, less than 8% (Table 1). Nevertheless, no lactate was detected during the enrichment process. *Clostridium muellerianum*, which constituted about 0.1% of the population, is a recently isolated CO-oxidizing acetogen (Doyle et al., 2022). It can produce acetate, butyrate, caproate, ethanol, and hexanol from CO and CO_2_ (Doyle et al., 2022). Considering the low relative abundance (<0.1%), the actual pH = 5, and its pH range for growth (pH 5.0–8.5) (Doyle et al., 2022), it is unlikely to have contributed to the metabolite production to any major extent. Therefore, the bacterial community other than *C. autoethanogenum* is assumed to have persisted because of growth on the yeast extract present in the culture medium. In conclusion, the bacterial community analysis showed that the CO-oxidizing bacterium *C. autoethanogenum* (or *C. ljungdahlii*) was well enriched and become the dominant species under the given condition.

**Table 1:** 16S rRNA microbial community analysis using the V1–V2 and V3–V4 primer sets.

	V1–V2 (%)	V3–V4 (%)
*Clostridium autoethanogenum*/*Clostridium ljungdahlii*	89.14	94.32
*Lactocaseibacillus paracasei*	7.49	–
*Lactocaseibacillus chiayiensis*	–	4.03
*Enterococcus hirae*	1.80	1.07
*Caproicibacter fermentans*	1.07	0.32
*Caldanaerobius fijiensis*	–	0.17
*Clostridium muellerianum*	–	0.05
*Clostridium neuense*	0.04	–
*Massiliimalia massiliensis*	0.04	–
*Ethanoligenens harbinense*	0.04	0.00
*Caproiciproducens galactitolivorans*	–	0.02
*Enterococcus thailandicus*	–	0.01

### 3.2 Effect of electrical current on CO_2_ electro-fermentation

Since *C. autoethanogenum* (or *C. ljungdahlii*) was highly predominant in the CO-enriched mixed culture, the operating conditions for CO_2_ electro-fermentation was chosen according to a previous microbial electrosynthesis study using *C. ljungdahlii*, in which it was found that the optimal cathodic potential was between −0.8 V and −1.0 V (Im et al., 2022). Therefore, 10 mA and 25 mA of fixed current were chosen, which corresponded to −0.8 V and −1.0 V in the results of the previous study (Im et al., 2022).

**Figure 1:**
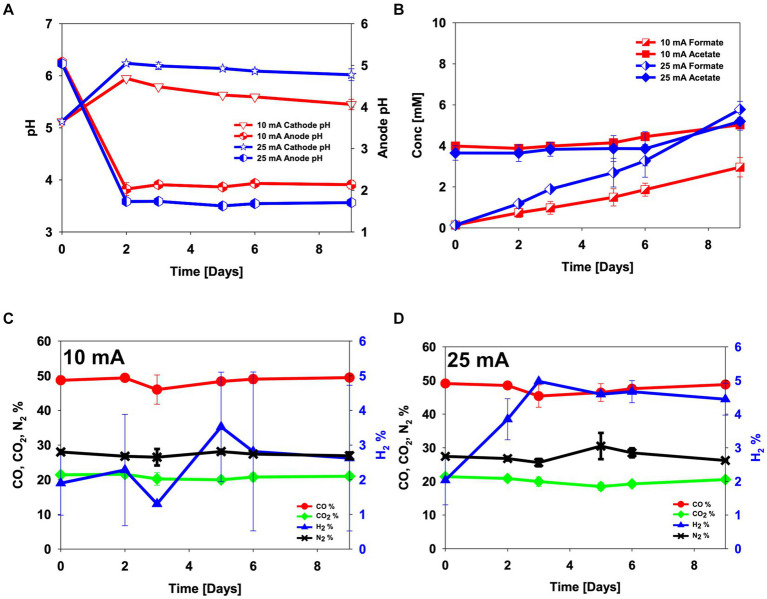
Abiotic control experiment in electrochemical H-type reactors sparged with 50% CO, 20% CO_2_ and 30% N_2_. **(A)** pH, **(B)** metabolite concentrations, **(C,D)** headspace gas composition in abiotical reactors operated at **(C)** 10 mA and **(D)** 25 mA. Error bars indicate standard deviation (*n* = 2).

The electro-activity of the enriched culture was first tested in CO_2_ electro-fermentation. An abiotic experiment was conducted as a control. Electrical current levels of 10 mA and 25 mA were chosen to test the electro-activity. The abiotic experiment was done by sparging a gas mix containing 30% N_2_, 50% CO and 20% CO_2_ to test if the fixed electric current applied to each reactor ensured constant hydrogen evolution from the electrode and to estimate if changes in hydrogen partial pressure could be used as an indicator of electro-activity (Figure 1). Throughout the experiments, hydrogen was detected in the headspace of the abiotically operated reactors, at 1.5–3.5% when 10 mA was applied to the reactor and 2–5% when 25 mA was applied (Figures 1C,D). While no acetate production was observed in the abiotic experiment, formate was electrochemically produced (3.0 ± 0.5 mM for 10 mA and 5.8 ± 0.4 mM for 25 mA, *p*-value = 0.03) (Figure 1B).

**Figure 2:**
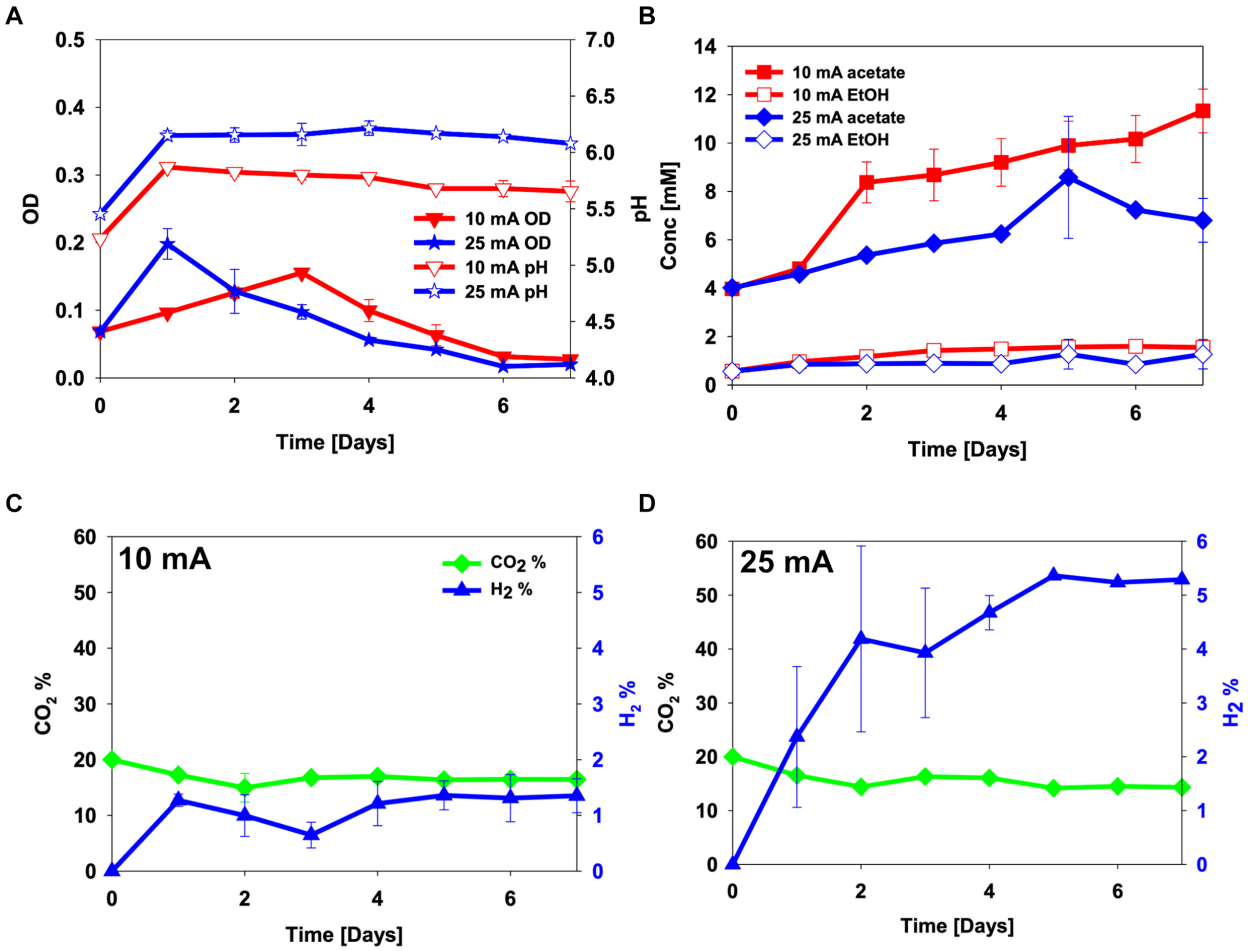
CO_2_ electro-fermentation using the enriched culture in H-type reactors sparged with 20% CO_2_ and 80% N_2_. **(A)** pH and optical density (OD), **(B)** metabolite concentrations, **(C,D)** headspace gas composition in reactors operated at **(C)** 10 mA and **(D)** 25 mA. Error bars indicate standard deviation (*n* = 2).

To assess the electro-activity of the enriched culture on CO_2_, H-type BES reactors were sparged with a gas mix of 20% CO_2_ and 80% N_2_, and were inoculated with the enriched culture. As in the abiotic experiment, the pH increased after the first day from an initial pH 5.0 to pH 5.9 and pH 6.2 for 10 mA and 25 mA reactors, respectively. After this, acetate started being produced, and the pH was maintained in the 25 mA reactor and gradually decreased with time in the 10 mA reactor (Figure 2A). At the end of the experiments, concentrations of 11.3 ± 0.9 mM acetate and 1.6 ± 0.1 mM ethanol were detected in the 10 mA reactor, and 6.8 ± 0.9 mM of acetate and 1.2 ± 0.6 mM of ethanol in the 25 mA reactor (Figures 2A,B). The 10 mA reactor showed some cell growth until day 3, after which the cell growth decreased. Hydrogen in the headspace slightly decreased to 0.6% ± 0.2% until day 3, after which it increased again to around 1.3% ± 0.3% on day 5. The cell growth rate in the 25 mA reactor was faster (*μ* = 0.044 ± 0.001 h^−1^) than that in the 10 mA reactor (*μ* = 0.011 ± 0.001 h^−1^) (*p*-value = 0.01). In the 25 mA reactor, the OD was highest on day 1, after which it decreased. The OD in the 10 mA reactor gradually increased through day 3, after which it decreased. The percentage of hydrogen in the headspace instead increased to become similar to the abiotic experiment (Figure 2).

**Figure 3:**
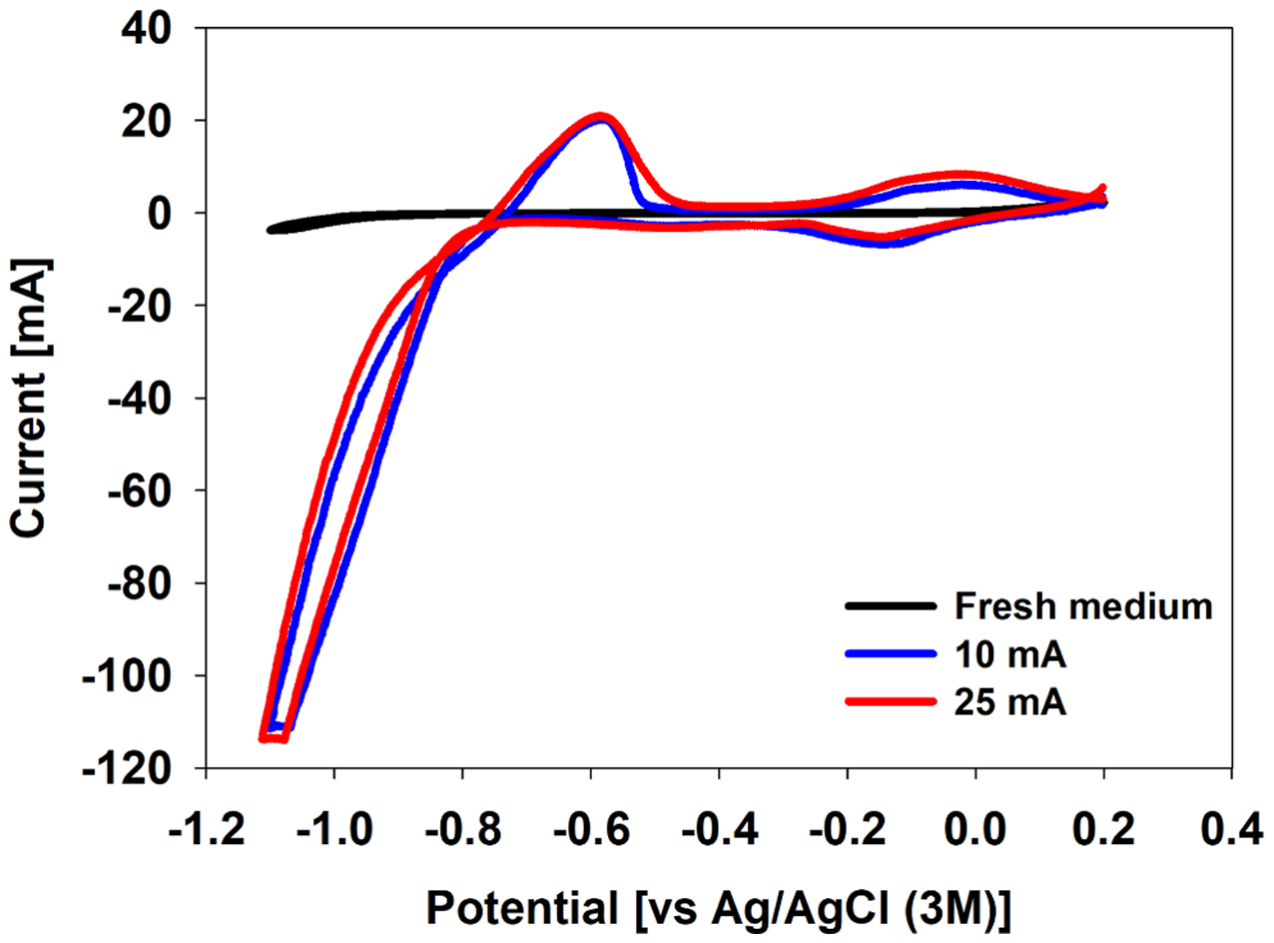
Cyclic voltammogram of fresh medium and culture media at the end of the biotic experiments under CO_2_ condition in a BES reactor.

At the end of the experiment, cyclic voltammetry was performed to identify extracellular electron transfer to the culture (Figure 3). While no significant redox activities were observed with fresh medium without inoculum, both the 10 mA and the 25 mA reactors showed a drastic reductive current increase below −0.8 V before an oxidative peak around −0.6 V (Figure 3). This indicates improved catalysis of the hydrogen evolution reaction on the cathode.

### 3.3 Effect of electrical current on CO electro-fermentation

**Figure 4:**
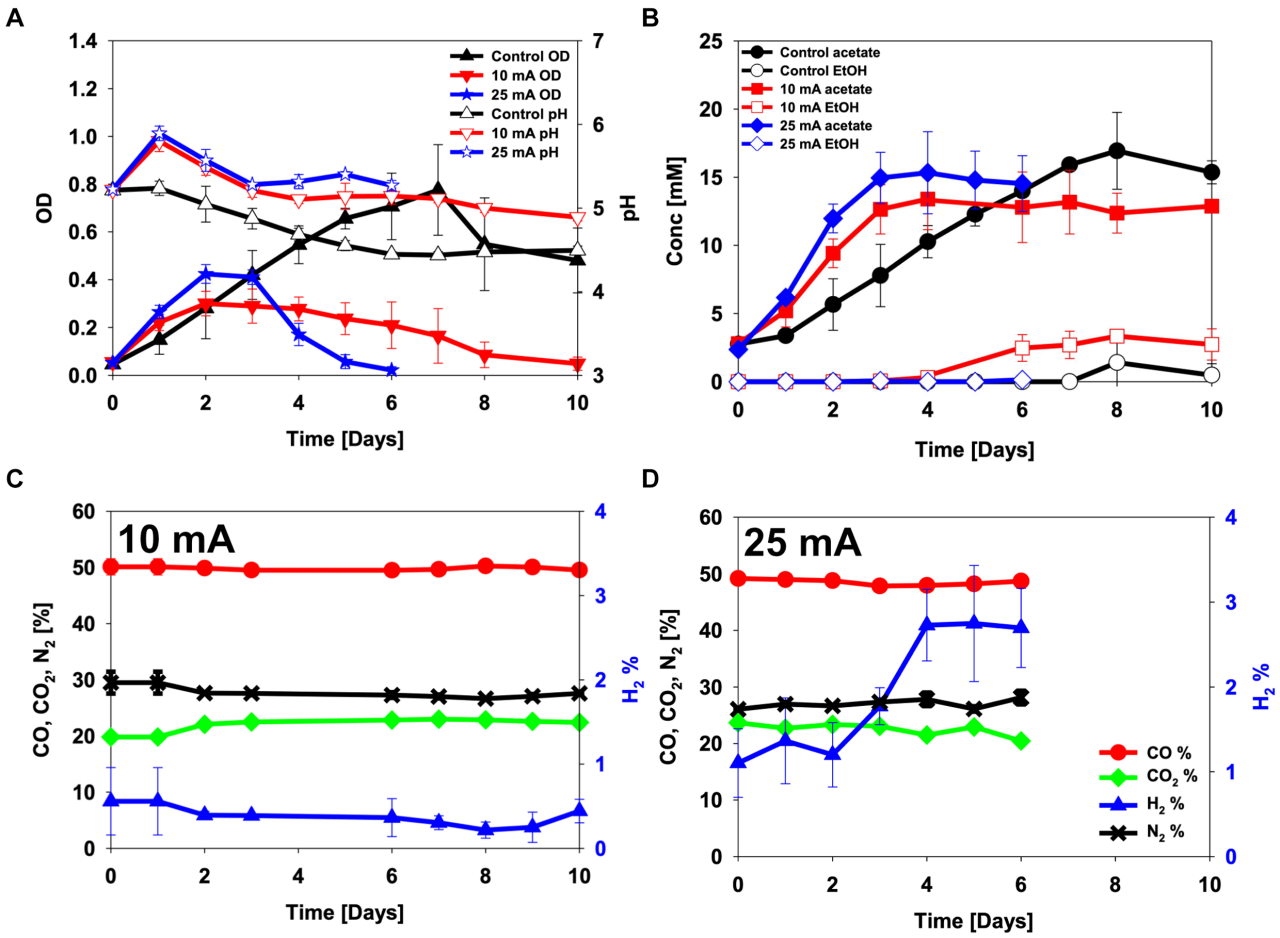
CO electro-fermentation using the enriched culture in H-type reactors sparged with 50% CO, 20% CO_2_ and 30% N_2_. **(A)** pH and optical density (OD), **(B)** metabolite concentrations, **(C,D)** headspace gas composition in reactors operated at **(C)** 10 mA and **(D)** 25 mA. Error bars indicate standard deviation (*n* = 3).

The enriched bacterial culture was inoculated in an H-type BES reactor to establish if applying an electric current could change the metabolite profile during CO electro-fermentation without an electron mediator. As a control experiment, no electric current was applied to the reactor. When no electric current was applied, the maximum OD, 0.78 ± 0.19, was achieved on day 7, when 16.9 ± 2.8 mM of acetate was detected (Figures 4A,B). Only one of the triplicate experiments produced 4.2 mM of ethanol, while the other two produced no ethanol.

The OD increased faster in both the 10 and the 25 mA reactors than in the control during the first day, but after day 2 it started to decrease. When 10 mA of electric current was applied, the maximum OD was lower (0.30 ± 0.05) than in the control experiment (0.78 ± 0.19) (Figure 4A). At the end of the experiment, 12.9 ± 2.7 mM acetate and 2.7 ± 1.1 mM ethanol were detected in the 10 mA reactor (Figure 4B). The H_2_ content in the headspace gradually decreased from 0.6% ± 0.4% on day 1 to 0.2% ± 0.1% on day 8, indicating that the enriched inoculum was utilizing hydrogen during CO electro-fermentation (Figure 4C). When an electric current of 25 mA was applied, the initial cell growth and acetate production became even faster than when 10 mA current was applied to the BES reactor. The maximum OD reached 0.42 ± 0.04 on day 2, and 15.3 ± 3.0 mM of acetate was detected on day 4 (Figures 4A,B). The OD decreased from day 3 onwards, which corresponded to an increase in the hydrogen content in the headspace (Figure 4D). Overall, this experiment showed that the CO-oxidizing bacterial culture could utilize electrons from the electrode for acetate production and cell growth during CO electro-fermentation (Figure 4). Nonetheless, the applied current caused a decrease in the OD, presumably due to cell death (Figure 4).

**Figure 5:**
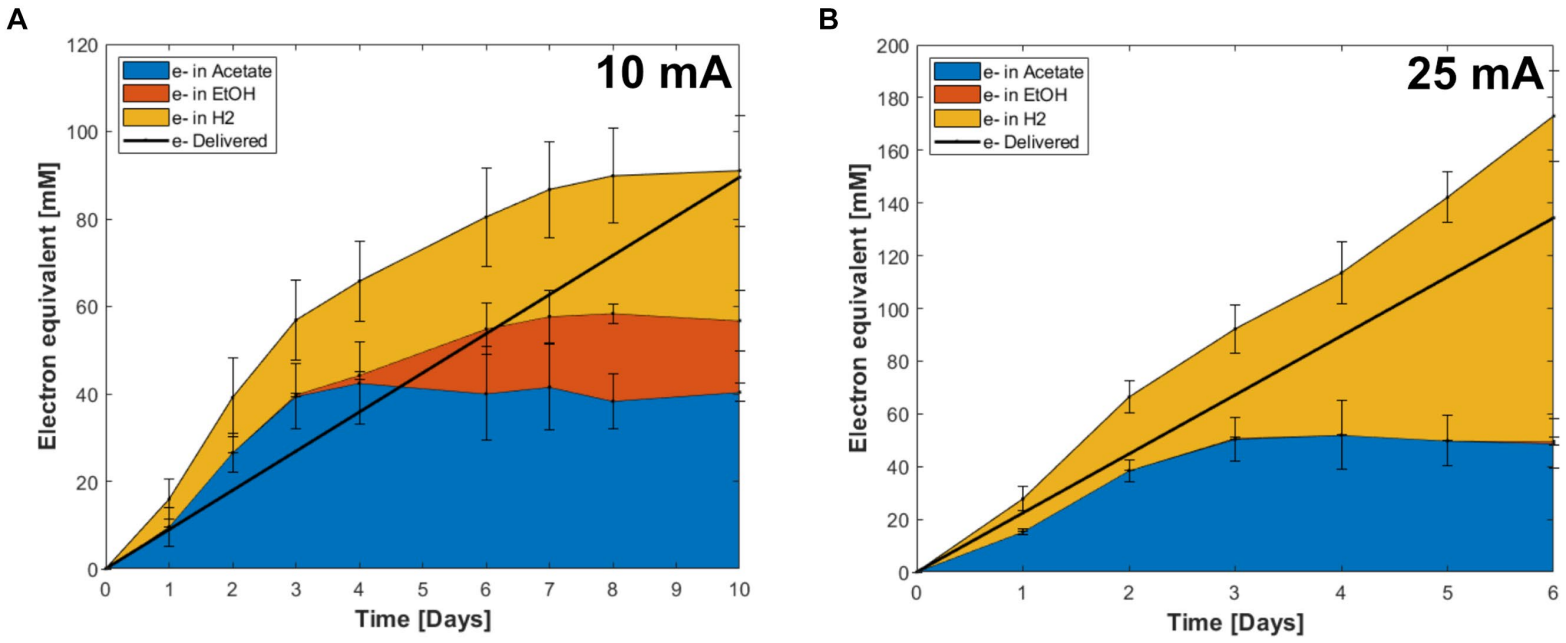
Electron recovery into metabolites in CO electro-fermentation using the enriched culture in reactors supplied with **(A)** 10 mA and **(B)** 25 mA. Error bars indicate standard deviation (*n* = 3).

Coulombic efficiency is an important parameter in the evaluation of electro-fermentation processes, since it indicates the recovery of energy, supplied by the electrode, in the metabolic products. The CEs varied depending on the stage of the fermentations (Figure 5). Both the 10 mA and 25 mA reactors produced acetate and hydrogen as the main products until day 3, when the highest CE and OD were measured. The highest CE observed at day 3 from the 10 mA applied reactor was 148% ± 29% when acetate and ethanol were considered, and 212% ± 40% when also including H_2_ (Figure 5). At 25 mA the corresponding numbers were 75% ± 11 and 120% ± 31%, respectively (Figure 5). After day 3, acetate production ceased, and most of electrons were recovered in H_2_ along with the decrease in OD (Figures 4, 5).

## 4 Discussion

*Clostridium autoethanogenum* is a well-known ethanol producer during CO and CO_2_ fermentation (Marcellin et al., 2016; Heffernan et al., 2020). *C. autoethanogenum* and *C. ljungdahlii* are closely related and are often mentioned together when syngas fermentation is discussed (Brown et al., 2014). Although genomic and phenotypic differences of the two strains have been reported (Cotter et al., 2009a,b), the two strains are similar enough to be indistinguishable at the 16S rRNA level, and the overall similarity of the two suggests they share a common ancestor (Stackebrandt et al., 1999; Brown et al., 2014). The electro-activity of *C. ljungdahlii* has been studied since the beginning of microbial electrosynthesis research (Nevin et al., 2011; Bajracharya et al., 2015; Wang et al., 2020; Zhu et al., 2022). Although the electro-activity of *C. autoethanogenum* has not been tested under autotrophic conditions, its electro-activity has been demonstrated under heterotrophic conditions both with and without an electron mediator (Kracke et al., 2016; Martínez-Ruano et al., 2023).

CO_2_ electro-fermentation using the enriched culture at 25 mA showed lower acetate production (*p*-value = 0.04) than with 10 mA while higher and faster cell growth was seen in the reactor at 25 mA on day 1 (Figures 2A,B). This might be attributed to the drastic pH increase (pH > 6.0), as the optimal pH for growth of *C. autoethanogenum* and *C. ljungdahlii* is pH 6.0 (Figure 2A) (Tanner et al., 1993; Abrini et al., 1994). The cell potentials during CO_2_ electro-fermentation at 10 mA and 25 mA were gradually decreased from −3.9 V to −3.5 V and from −5.5 V to −4.7 V, respectively (Supplementary material). The decrease in the cell potentials may be due to improved catalysis for H_2_ evolution of the culture in the BES reactor. No visible biofilm was observed on the cathodes. The cyclic voltammetry showed that current consumption started at around −0.75 V and showed a drastic increase in current consumption between −0.8 V and −1.1 V (Figure 3). Similar cyclic voltammetry trends as those observed in this study have previously been observed with *Clostridium ljungdahlii*, which was claimed to utilize hydrogen-mediated external electron transfer (Figure 3) (Im et al., 2022; Boto et al., 2023). The OD decreased already after day 1 and 3 (Figure 2A). If the decrease in OD was because of biofilm formation and a biofilm of the enriched culture had an important role for extracellular electron transfer in this system, the CV would have shown another reductive peak between −0.6 and −0.8 V, or a curved change below −0.8 V (Figure 3). When Nevin et al. tested microbial electrosynthesis using *C. ljungdahlii*, a [N_2_:CO_2_:H_2_] = [83:10:7] gas mix was used to promote biofilm formation (Nevin et al., 2011). However, scanning electron microscopy and confocal laser-scanning microscopy results showed only a thin layer of active cells on the electrode (Nevin et al., 2011). Biofilm formation of *C. ljungdahlii* can be induced by sodium chloride stress (Philips et al., 2017). However, multiple batch operations of microbial electrosynthesis using *C. ljungdahlii* with electrochemically produced H_2_ demonstrated the importance of planktonic cells, with non-sustainable performances after multiple medium replacements (Bajracharya et al., 2015). Considering current densities used in this study were much higher (40 and 100 mA/L) than in the microbial electrosynthesis study using *C. ljungdahlii* (estimated volumetric current density ~ 7.5 mA/L) (Bajracharya et al., 2015), the planktonic cells were assumed to play a dominant role in this study. Altogether, the enriched culture could utilize electrons from the electrode and produce acetate in the same way as *C. ljungdahlii* utilizes electrons from an electrode. Given the uncertainty whether the main microorganism in the enriched culture was *Clostridium ljungdahlii* or *Clostridium autoethanogenum*, the results either confirm observations for *Clostridium ljungdahlii*, or show that *Clostridium autoethanogenum* also possesses electro-activity.

Electro-fermentation of CO has previously been done with undefined microbial consortia and single cultures, with or without the addition of electron mediators (Table 2). The necessity of using an exogenous electron mediator in previous studies was assumed to be due to the inhibitory effect of CO on electron transfer (Im et al., 2018; Chu et al., 2020). Carbon monoxide is a well-known inhibitor of hydrogenase and heme proteins, that acts by binding Fe^II^ (Purec et al., 1962; Wilson et al., 2012). Hence, consumption of H_2_ produced from the cathode is challenged in the presence of CO. CO electro-fermentation was previously tested with an electron mediator to bypass the inhibition on extracellular electron transfer (Im et al., 2018). When an electron mediator was used for CO electro-fermentation, a theoretical coulombic efficiency of about 200% was easily achieved (Im et al., 2018), while CEs of CO electro-fermentation without an electron mediator were so far reported to be lower than 100% (Table 2) (Chu et al., 2020). On the contrary, the results from this study suggest that utilization of electrons transferred from the electrode during CO electro-fermentation is possible as long as the cell viability is maintained and the inoculum is well enriched with CO-oxidizing acetogens (Figure 4). When CO-oxidizing acetogens are well enriched in the culture, the concentration of dissolved CO would stay low, since CO would be consumed by the CO-oxidizing acetogens. Thus, the inhibitory effect of CO on extracellular electron transfer may be minimized.

**Table 2:** Performance comparison of reported CO electro-fermentation trials.

Inoculum	Gas composition	Operational condition [Volumetric current density]^a^	Cathode material	Products	Acetate production rate (g/L/day)	Acetate and ethanol titers (g/L)	Coulombic efficiency (%)	Note	References
Anaerobic digester sludge	[N_2_:CO:CO_2_] = [50:40:10]	−1.1 V (vs Ag/AgCL) [16–32 mA/L]	Graphite felt	Acetate, Propionate, Butyrate, Isobutyrate, Isovalerate	0.34	Ac 8.60 ± 0.15	>200	Neutral Red used as an electron mediator	Im et al. (2018)
Mixed sediment	[N_2_:CO:CO_2_] = [50:40:10]	−1.1 V (vs Ag/AgCL), +yeast extract [160–532 mA/L]	Graphite felt	Acetate, Propionate, Butyrate, Isobutyrate, Isovalerate	0.17	Ac 3.5 ± 0.1	~190	HNQ^b^ used as an electron mediator	Song et al. (2020)
Mixed sediment	[N_2_:CO:CO_2_] = [50:40:10]	−1.1 V (vs Ag/AgCL) [264 mA/L]	Carbon felt	Acetate, Propionate, Butyrate, Isobutyrate, Isovalerate	0.71	Ac 6.89	184	HNQ^b^ used as an electron mediator	Song et al. (2021)
Activated sludge	[CO:CO_2_] = [0:100]	10 A m^−2^ [74 mA/L]	Carbon felt	Acetate, Butyrate, Caproate	–	Ac 4.84 ± 1.21	85 ± 3		Chu et al. (2020)
	[CO:CO_2_] = [25:75]			Acetate, Butyrate, Caproate	–	Ac 6.44 ± 0.05	76 ± 3		
	[CO:CO_2_] = [50:50]			Acetate, Butyrate, Caproate	–	Ac 5.47 ± 0.10	43 ± 1		
	[CO:CO_2_] = [75:25]			Acetate, Butyrate, Caproate	–	Ac 5.34 ± 0.20	51 ± 6		
*Clostridium ljungdahlii*	100% CO_2_	−1.8 V (vs Ag/AgCL) [640–820 mA/L]	2D electrode	Acetate, ethanol	–	Ac 4.9 Eth 1.2	Ac 17 ± 5 Eth 3 ± 2	The electrode produces CO from CO_2_ by electrochemical conversion, YTF medium^c^	Zhu et al. (2022)
		−1.2 V (vs Ag/AgCL) [440–980 mA/L]	3D electrode	Acetate, ethanol	–	Ac 6.0 Eth 1.4	Ac 30 ± 10 Eth 9 ± 8		
Cow fecal waste enriched under CO	[N_2_:CO:CO_2_] = [30:50:20]	10 mA [40 mA/L]	Graphite felt	Acetate, ethanol	0.10^d^	Ac 0.60 ± 0.03 Eth 0.12 ± 0.05	148 ± 29		This study
		25 mA [100 mA/L]		Acetate	0.12^d^	Ac 0.72 ± 0.14	75 ± 11^c^		

a The values for current are estimated values from the references in which potentiostatic operation was used.

b 2-hydroxy-1,4-naphthoquinone.

c Rich culture broth consisting of yeast extract-tryptone-fructose.

d The values were calculated during the exponential phase (until day 3).

The weak hydrogenase inhibition is illustrated by the H_2_ in the headspace of both the 10 mA and 25 mA reactors, which was lower than the H_2_ evolution obtained in the abiotic control experiment (*p*-value = 0.02 and 0.01, respectively). Moreover, the hydrogen content increased at the end of the experiment as the OD decreased (Figure 4). This clearly indicates microbial consumption of the electrochemically generated hydrogen. It should be noted that the H-type BES reactor used in this study was not designed to improve mass transfer of gas to liquid to compensate for the low solubility of CO and H_2_. This likely explains why CO consumption was not that noticeable during headspace analysis using GC (Figures 4C,D). The medium used here contained 1 g/L of yeast extract, but previous studies have shown that metabolite production from yeast extract without an electron source is negligible (Im et al., 2022).

Higher concentration of alcohol production has always been the focus of syngas fermentation. To achieve this, many different approaches have been introduced, such as modifications of medium composition, culture techniques, and cultivation temperatures (Abubackar et al., 2015; Phillips et al., 2015; Ramio-Pujol et al., 2015; Monir et al., 2020). The application of electrical current can be another approach to increase alcohol production. The control reactor without applied current produced 28.0 ± 9.4 mM/OD acetate and 2.5 ± 2.5 mM/OD ethanol, over the whole experiment. Interestingly, when 10 mA was applied to the reactor, the acetate production was 300 ± 200 mM/OD acetate and the ethanol production was significantly increased to 62.4 ± 17.5 mM/OD (*p*-value = 0.02) (Figures 4A,B). A similar result was observed in a previous study, when H_2_ was supplied with CO (Valgepea et al., 2018). In the study, [CO:Ar] = [60:40] condition, [CO:H_2_:CO_2_:Ar] = [50:20:20:10] condition, and [CO:H_2_:Ar] = [15:45:40] condition were compared to see the effect of H_2_ supplementation on gas fermentation (Valgepea et al., 2018). Supplementation of H_2_ significantly decreased the production of CO_2_ as by-product and increased carbon flux to ethanol by a factor four (Valgepea et al., 2018). This may be explained by the fact that H_2_ oxidation leads to ferredoxin reduction for ethanol production and NADH formation for CO_2_ reduction, while CO oxidation leads only to ferredoxin reduction.

Traditional bioreactors require delicate control of operational parameters such as temperature, pH, substrate availability, nutrient level, dissolved gas levels (pO_2_ or pCO_2_) for desirable performance (Asimakopoulos et al., 2018). Operation of electro-fermentation processes need control of more operational parameters because current flow in bioreactors changes the distribution of ions across the culture broth as the electrode is polarized (Harnisch and Schroder, 2009). An ion exchange membrane is introduced to maintain equal ion concentrations in each anode and cathode chamber. However, the mobility of protons and hydroxide ions in an ion exchange membrane is usually slower than in an aqueous solution, and slower than the formation of protons at the anode and the reduction of protons at the cathode (Harnisch et al., 2009; Harnisch and Schroder, 2009). This imbalance between the mobility and consumption of protons leads to an increase in pH in the cathode chamber, with the extent of the pH increase varying based on the current flow (Im et al., 2022). The extent of the pH increase might influence ethanol production as well as cell viability during CO electro-fermentation. Ethanol production from CO by *C. ljungdahlii* and *C. autoethanogenum* mainly occurs via aldehyde:ferredoxin oxidoreductase- (AOR-) mediated conversion of acetaldehyde, which is formed from intracellular acetate. The ratio between the undissociated and dissociated forms of an acid varies depending on the pH, and only the undissociated acetic acid can diffuse over the cell membrane (Richter et al., 2016; Valgepea et al., 2018). At high extracellular pH, acetate would primarily be present in its dissociated anion form, which would lead to a depletion of intracellular acetate and low acetaldehyde formation. This would effectively hinder ethanol formation. The bulk pH observed in both 10 mA and 25 mA reactors were not significantly different (Figure 4A). It would be difficult to measure the local pH around the electrodes. However, it is likely that the local pH at the surface of a cathode supplying 25 mA is much higher than when 10 mA is supplied, as the extent of reduction of protons, the primary electron acceptor, to H_2_ would be higher in the 25 mA case. Hypothetically, such local high pH would stop ethanol production at higher currents despite the supply of reducing power.

Likewise, when an electric current was applied to the reactor, inhibition in cell growth was observed (Figure 4). The maximum OD of the culture supplied with 10 mA was lower than that of the control (*p*-value = 0.04) and of the 25 mA-supplied culture (*p*-value = 0.02), but the decrease in OD was slower than that of the control and the 25 mA reactor (Figure 4). Low cell growth during microbial electrosynthesis using *C.ljungdahlii* has been reported several times (Im et al., 2022; Boto et al., 2023). It has been speculated that this is because of limited electron supply from the electrode or elevated pH around the electrode (Im et al., 2022). In our experiments, CO was available as the primary carbon and electron source, in addition to CO_2_ and electrons from the electrode. Therefore, low cell growth of *C. ljungdahlii* in a BES was not due to limited electron supply from the electrode. Indeed, the current supply was helping the cell grow and acetate production for the first 2 days (Figure 4). Therefore, the problem might be elevated pH at the cathode.

Different initial pH for microbial electrosynthesis using *C. ljungdahlii* was not previously reported, but different initial pH for autotrophic growth of *C. ljungdahlii* has been tested (Cotter et al., 2009a; Im et al., 2022). In the studies by Im et al., and Cotter et al., the focus was on the effect of pH on ethanol production from gas fermentation, but *C. ljungdahlii* only showed improved cell growth and acetate production at initial pH 5.0 (Cotter et al., 2009a; Im et al., 2022). The authors tested microbial electrosynthesis using *C. ljungdahlii* at initial pH 5.0 to have better acetate production and to mitigate elevated pH around the electrode (Im et al., 2022). However, Boto et al., suggested high inoculation cell density could achieve a continuous increase in the OD (Boto et al., 2023). This might suggest a quorum sensing system involved for planktonic cell growth during microbial electrosynthesis (Piatek et al., 2022).

This study investigated a CO-enriched culture in a bioelectrochemical system using CO_2_ and CO as carbon sources. The enriched culture was dominated by *C. autoethanogenum* or *C. ljungdahlii*. A bioelectrochemical system could affect the metabolite profile during CO electro-fermentation. The coulombic efficiency is used to evaluate the efficiency of BES processes (Jourdin and Burdyny, 2021). The CE indicates how many electrons are recovered in metabolites compared to the number of electrons input to the system. CEs of CO_2_ electro-fermentation usually stay around 100% or less because the electrode is the main electron donor for CO_2_ reduction (Im et al., 2022). However, in CO electro-fermentation, where CO is used as the primary electron donor and carbon source, the aim is to achieve more than 100% of CE by re-utilizing CO_2_ generated from CO oxidation (Im et al., 2018). In this study, CEs of acetate and ethanol production was 148% ± 29% when applying 10 mA to the BES, indicating an efficient electron uptake. At 10 mA, the culture was able to produce more ethanol during CO electro-fermentation than at 25 mA. These results suggest that appropriately designed CO electro-fermentation can increase the yield of a targeted chemical using industrial waste gas, and enhance the performance of a bioelectrochemical system.

## Data availability statement

The 16S rRNA sequencing data presented in this study are deposited in NCBI Sequence Read Archive (GenBank), with accession numbers PQ164455 - PQ164475. All data is also available in the Supplementary material.

## Author contributions

CI: Writing – original draft, Visualization, Methodology, Investigation, Formal analysis, Conceptualization. MK: Writing – review & editing, Methodology, Formal analysis. JRK: Writing – review & editing, Funding acquisition. KV: Writing – review & editing, Visualization. OM: Writing – review & editing, Supervision, Conceptualization. YN: Writing – review & editing, Supervision, Funding acquisition. CJF: Writing – review & editing, Supervision, Project administration.
