# Supplementary material for: Low electric current in a bioelectrochemical system facilitates ethanol production from CO using CO-enriched mixed culture
Source: Front Microbiol. 2024 Aug 29;15:1438758. doi: 10.3389/fmicb.2024.1438758 (PMC11390636; doi:10.3389/fmicb.2024.1438758)
Supplement: Supplementary file 1 [file Data_Sheet_1.ZIP › 0. List of contents.docx]

**Supplementary information**

**Contents**

1. Cell potentials.xlsx

Cell potentials data measured during galvanostatic operation for CO_2_ and CO electro-fermentation.

1. 16s rRNA sequencing.zip

Raw data from 16s rRNA sequencing of CO-enriched mixed culture as provided by the sequencing service.

1. Abiotic CO experiment.xlsx

Abiotic experiment data containing GC, HPLC, and OD measurements.

1. CO2 electro-fermentation.xlsx

CO2 electro-fermentation experiment data containing GC, HPLC, and OD measurements.

1. Cyclic voltammetry results.xlsx

Cyclic voltammetry results measured after CO_2_ electro-fermentation.

1. 0 mA biotic CO electro-fermentation.xlsx

0 mA CO electro-fermentation experiment data containing GC, HPLC, and OD measurements.

1. 10 mA biotic CO electro-fermentation.xlsx

10 mA CO electro-fermentation experiment data containing GC, HPLC, and OD measurements.

1. 25 mA biotic CO electro-fermentation.xlsx

25 mA CO electro-fermentation experiment data containing GC, HPLC, and OD measurements.

1. Cell growth rate calculation.xlsx

Cell growth rate data calculated for all electro-fermentation experiments.
