# Supplementary material for: Low electric current in a bioelectrochemical system facilitates ethanol production from CO using CO-enriched mixed culture
Source: Front Microbiol. 2024 Aug 29;15:1438758. doi: 10.3389/fmicb.2024.1438758 (PMC11390636; doi:10.3389/fmicb.2024.1438758)
Supplement: Supplementary file 1 [file Data_Sheet_1.ZIP › 2. 16s rRNA sequencing.zip!/16s rRNA sequencing/HN00197155_OTU_2/OTUanalysis.html]

NGS Analysis Report


# NGS Analysis Report

Info

|  |  |  |  |  |
| --- | --- | --- | --- | --- |
|  | Company / Institution | Macrogen Europe | Target Region(Primer Set) | V3V4(Bakt\_341F-805R) |
|  | Customer | Macrogen Europe | Length Filter(Assembled Read) | 400bp <= Good Sequences <= 500bp |
|  | Order Number | HN00197155 | OTU Picking Method(Tool) | De novo(CD-HIT-OTU) |
|  | Number of Samples | 1Sample | DB Version(Tool) | NCBI\_16S\_20230103(BLAST) |

## I. Analysis Procedure

**MANUAL Download**


Preprocessing&Clustering


Taxonomic assignment


Diversity statistics

## II. Results of Analysis

Results of Analysis

|  |  |
| --- | --- |
| Assembly | Results of Assembly [ HTML ] [ EXCEL ] |
| Preprocessing & Clustering | Summary [ HTML ]  Representative sequence (OTU) [ FASTA ] |
| Taxonomic assignment - blast\_NCBI\_16S | Bar type [ HTML ]  OTU abundance [ EXCEL ] / Results of BLAST [ EXCEL ]   Taxonomy abundance - Count [ EXCEL ] / Ratio [ EXCEL ] |
| Diversity statistics | Community richness & diversity [ HTML ] [ EXCEL ]  Rarefaction [ HTML ] |

Info2


| The data retention period is three months,  please send an email(ngs@macrogen.com) or contact representative  if you want longer retention period. | Macrogen Korea | Contact | |  |
|  | 10F, 254 Beotkkot-ro, Geumcheon-gu, Seoul Rep. of Korea Phone: +82-2-2113-7000 |  | Web : ngs@macrogen.com Lims : https://dna.macrogen.com |
