# Supplementary material for: Low electric current in a bioelectrochemical system facilitates ethanol production from CO using CO-enriched mixed culture
Source: Front Microbiol. 2024 Aug 29;15:1438758. doi: 10.3389/fmicb.2024.1438758 (PMC11390636; doi:10.3389/fmicb.2024.1438758)
Supplement: Supplementary file 1 [file Data_Sheet_1.ZIP › 2. 16s rRNA sequencing.zip!/16s rRNA sequencing/HN00197155_OTU_2/OTU_Results/STAT.html]

Results of Assembly


# NGS Analysis Report - OTU

## Results of Assembly (by FLASH)

| Read Count by Sample | | | | | | |
| --- | --- | --- | --- | --- | --- | --- |
| | Read Quality by Sample | | | | | | | | --- | --- | --- | --- | --- | --- | --- | | | | | | | | | | Results of Assembly (by FLASH)          | SampleName | Total Bases | Read Count | N(%) | GC(%) | Q20(%) | Q30(%) | | COenrichment.V4 | 78,621,325 | 178,091 | 0.0001 | 52.62 | 98.83 | 95.39 | | \* Total Bases : The total number of bases in reads indentified  \* Read Count : The total number of sequence reads  \* N(%) : The N percentage in sequence reads  \* GC(%) : The GC percentage in sequence reads  \* Q20(%) : The percentage of bases in which the phred score is above 20  \* Q30(%) : The percentage of bases in which the phred score is above 30 | | | | | | | | | | | | | | |
