# Supplementary material for: Low electric current in a bioelectrochemical system facilitates ethanol production from CO using CO-enriched mixed culture
Source: Front Microbiol. 2024 Aug 29;15:1438758. doi: 10.3389/fmicb.2024.1438758 (PMC11390636; doi:10.3389/fmicb.2024.1438758)
Supplement: Supplementary file 1 [file Data_Sheet_1.ZIP › 2. 16s rRNA sequencing.zip!/16s rRNA sequencing/HN00197155_OTU_2/OTU_Results/otu_table_summary.html]

Summary


# NGS Analysis Report - OTU

## Summary - Preprocessing & Clustering (by CD-HIT-OTU)

## OTU Picking Method: de novo

| - Results of Clustering (cutoff : 97%) | | ||| No. | SampleName | Read Count |
| --- | --- | --- |
| 1 | COenrichment.V4 | 73,849 |

| - Results of Preprocessing | |
| --- | --- |
| Sample Count | 1 |
|  | |
| Read Count | 73,849 |
|  | |
| Gamma-diversity | 21 |
|  | |
| Counts/sample summary | |
| Min Max Median Mean | 73,849.0 73,849.0 73,849.0 73,849.0 |
|  | |
| Filtered Read Count | |
| Ambiguous Wrong prefix or primers (Sequence of prefix or primer) Low-Quality Chimera Other |  |

55  
8,906  
CCTACGGG[ACGT]GGC[AT]GCAG  
554  
5,234  
89,493

|  | |
