# Supplementary material for: Low electric current in a bioelectrochemical system facilitates ethanol production from CO using CO-enriched mixed culture
Source: Front Microbiol. 2024 Aug 29;15:1438758. doi: 10.3389/fmicb.2024.1438758 (PMC11390636; doi:10.3389/fmicb.2024.1438758)
Supplement: Supplementary file 1 [file Data_Sheet_1.ZIP › 2. 16s rRNA sequencing.zip!/16s rRNA sequencing/Raw_data/assets/spgs/aRq20Q30PopUp.html]

MACROGEN NGS Report


Q20/Q30 (%)

Total number of samples:

You can hover over the bars to see the data values.

Q20(%) 
 
Q30(%)

Q20(%) : 
Q30(%) :

TOP

Copyright © 
Research Use Only.
