# Supplementary material for: Low electric current in a bioelectrochemical system facilitates ethanol production from CO using CO-enriched mixed culture
Source: Front Microbiol. 2024 Aug 29;15:1438758. doi: 10.3389/fmicb.2024.1438758 (PMC11390636; doi:10.3389/fmicb.2024.1438758)
Supplement: Supplementary file 1 [file Data_Sheet_1.ZIP › 2. 16s rRNA sequencing.zip!/16s rRNA sequencing/Raw_data/assets/spgs/xResultFileDesc.html]

MACROGEN NGS Report


Order No.

- Workflow
  - Experimental Workflow
- Raw Data Result
  - Raw Data Statistics
  - Total Bases
  - GC/AT Content
  - Q20/Q30 (%)
  - Quality by Cycle
- Deliverables
  - Download List
- Appendix
  - FAQ
  - Result File Description


Research Use Only

Home

Appendix


- Workflow
- Raw Data Result
- Deliverables
- Appendix

Result File Description


- FAQ
- Result File Description

Result File Description

Deliverables List

|  |  |  |
| --- | --- | --- |
| File Type | File Name | Description |
| [Sample name]\_[read1].fastq.gz | Raw read1 sequence data |  |
| [Sample name]\_[read2].fastq.gz | Raw read2 sequence data |  |
| md5sum | [Order#]\_[#samples]\_md5sum\_[DownloadLink].txt | You can download this file by clicking on the "md5sum List" button found on the "Download List" page. The file is slightly different in terms content, depending on how you’re receiving your data. If you’re receiving via download link, the file contains the following information : File name, File size, md5sum, FTP link. Otherwise, if you're receiving your data via HDD the file only contains : File name, File size, and md5sum.    MD5 is a string of 32 hexadecimal values, which represents a 'fingerprint' of a file. By comparing the supplied MD5 value to the actual value computed by the MD5sums utility, you can make sure that the file that you downloaded off of the internet has not been tampered with or modified from the original file stored in our server. |

FASTQ Format

Phred Quality Score

Phred quality score numerically expresses the accuracy of each nucleotide. Higher Q number signifies higher accuracy.  
For example, if Phred assigns a quality score of 30 to a base, the chances of having base call error are 1 in 1000.  
Phred Quality Score Q is calculated with -10log_10_(P), where *p* is probability of erroneous base call.

|  |  |  |
| --- | --- | --- |
| Quality of phred score | Probability of incorrect base call | Base call accuracy |
| 10 | 1 in 10 | 90% |
| 20 | 1 in 100 | 99% |
| 30 | 1 in 1000 | 99.9% |
| 40 | 1 in 10000 | 99.99% |

Order Information

|  |  |
| --- | --- |
|  |  |
|  |  |
|  | Order Number |

The data can be downloaded

from the link below.


The download link is active for 2 weeks only, 
so please download your data within this period.
Once you download the data, please make sure to check the integrity
of the files. The sequencing files will be deleted from our server
3 months after the analysis report is released;
please contact us within 3 months if you encounter a problem with the data.


Download Data

Company location

Macrogen Gangnam HQ

Business & Support Center

Macrogen Bldg, 238, Teheran-ro,  
Gangnam-gu, Seoul, Republic of Korea  
Tel: +82-2-2180-7000  
Web : https://www.macrogen.com  
LIMS : http://dna.macrogen.com

Macrogen Genome Center

Laboratory, IT and Business

[08511] 1001, 10F, 254, Beotkkot-ro,  
Geumcheon-gu, Seoul, Republic of Korea  
(Gasan-dong, World Meridian 1)  
Tel: +82-2-2180-7000  
Email1: ngs@macrogen.com(Overseas)  
Email2: ngskr@macrogen.com  
(Republic of Korea)  
Web : https://www.macrogen.com  
LIMS : http://dna.macrogen.com

Psomagen
(Macrogen USA)

Laboratory, Business & Support Center

1330 Piccard Drive, Suite 103, Rockville,  
MD 20850, United States  
T. +1-301-251-1007  
Email : inquiry@psomagen.com  
Web : https://psomagen.com

Macrogen Japan

Laboratory, Business & Support Center

16F Time24 Building, 2-4-32 Aomi,  
Koto-ku, Tokyo 135-0064 JAPAN  
T. +81-3-5962-1124  
E. ngs@macrogen-japan.co.jp

Macrogen Singapore

Laboratory, Business & Support Center

3 Biopolis Drive #05-18, Synapse,  
Singapore 138623  
T. +65-6339-0927  
Email : info-sg@macrogen.com

Macrogen Europe

Laboratory, Business & Support Center

Meibergdreef 57, 1105 BA, Amsterdam,  
the Netherlands  
T. +31-20-333-7563  
Email : ngs@macrogen.eu

Macrogen Spain

Laboratory, Business & Support Center

Av. Sur del Aeropuerto de Barajas,  
28, Office B-2, 28042 Madrid, Spain  
T. +34-911-138-378  
Email : info-spain@macrogen.com

Macrogen Belgium

Laboratory, Business & Support Center

Oxfordlaan 70, 6229 EV Maastricht,  
Netherlands  
Tel: +31-20-333-7563  
Email : info.be@macrogen.eu

Macrogen Italy

Viale Ortles, 22/4, 20139 Milano,   
MI, Italy  
Tel: +39-02-5666-0274  
Email : italy@macrogen-europe.com

TOP

Copyright © 
Research Use Only.
