# Supplementary material for: Low electric current in a bioelectrochemical system facilitates ethanol production from CO using CO-enriched mixed culture
Source: Front Microbiol. 2024 Aug 29;15:1438758. doi: 10.3389/fmicb.2024.1438758 (PMC11390636; doi:10.3389/fmicb.2024.1438758)
Supplement: Supplementary file 1 [file Data_Sheet_1.ZIP › 2. 16s rRNA sequencing.zip!/16s rRNA sequencing/Raw_data/assets/spgs/aRgCATContent.html]

MACROGEN NGS Report


Order No.

- Workflow
  - Experimental Workflow
- Raw Data Result
  - Raw Data Statistics
  - Total Bases
  - GC/AT Content
  - Q20/Q30 (%)
  - Quality by Cycle
- Deliverables
  - Download List
- Appendix
  - FAQ
  - Result File Description


Research Use Only

Home

Raw Data Result


- Workflow
- Raw Data Result
- Deliverables
- Appendix

GC/AT Content


- Raw Data Statistics
- Total Bases
- GC/AT Content
- Q20/Q30 (%)
- Quality by Cycle

GC/AT Content

You can hover over the bars to see the data values.

GC(%) 
 
AT(%)

View All

GC: 

AT:

Order Information

|  |  |
| --- | --- |
|  |  |
|  |  |
|  | Order Number |

##### Information!

Your data will be retained in our server for 3 months. Should you wish to extend the retention period, please email or contact our sales team.

The data can be downloaded

from the link below.


The download link is active for 2 weeks only, 
so please download your data within this period.
Once you download the data, please make sure to check the integrity
of the files. The sequencing files will be deleted from our server
3 months after the analysis report is released;
please contact us within 3 months if you encounter a problem with the data.


Download Data

Company location

Macrogen Gangnam HQ

Business & Support Center

Macrogen Bldg, 238, Teheran-ro,  
Gangnam-gu, Seoul, Republic of Korea  
Tel: +82-2-2180-7000  
Web : https://www.macrogen.com  
LIMS : http://dna.macrogen.com

Macrogen Genome Center

Laboratory, IT and Business

[08511] 1001, 10F, 254, Beotkkot-ro,  
Geumcheon-gu, Seoul, Republic of Korea  
(Gasan-dong, World Meridian 1)  
Tel: +82-2-2180-7000  
Email1: ngs@macrogen.com(Overseas)  
Email2: ngskr@macrogen.com  
(Republic of Korea)  
Web : https://www.macrogen.com  
LIMS : http://dna.macrogen.com

Psomagen
(Macrogen USA)

Laboratory, Business & Support Center

1330 Piccard Drive, Suite 103, Rockville,  
MD 20850, United States  
T. +1-301-251-1007  
Email : inquiry@psomagen.com  
Web : https://psomagen.com

Macrogen Japan

Laboratory, Business & Support Center

16F Time24 Building, 2-4-32 Aomi,  
Koto-ku, Tokyo 135-0064 JAPAN  
T. +81-3-5962-1124  
E. ngs@macrogen-japan.co.jp

Macrogen Singapore

Laboratory, Business & Support Center

3 Biopolis Drive #05-18, Synapse,  
Singapore 138623  
T. +65-6339-0927  
Email : info-sg@macrogen.com

Macrogen Europe

Laboratory, Business & Support Center

Meibergdreef 57, 1105 BA, Amsterdam,  
the Netherlands  
T. +31-20-333-7563  
Email : ngs@macrogen.eu

Macrogen Spain

Laboratory, Business & Support Center

Av. Sur del Aeropuerto de Barajas,  
28, Office B-2, 28042 Madrid, Spain  
T. +34-911-138-378  
Email : info-spain@macrogen.com

Macrogen Belgium

Laboratory, Business & Support Center

Oxfordlaan 70, 6229 EV Maastricht,  
Netherlands  
Tel: +31-20-333-7563  
Email : info.be@macrogen.eu

Macrogen Italy

Viale Ortles, 22/4, 20139 Milano,   
MI, Italy  
Tel: +39-02-5666-0274  
Email : italy@macrogen-europe.com

TOP

Copyright © 
Research Use Only.
