# Supplementary material for: Low electric current in a bioelectrochemical system facilitates ethanol production from CO using CO-enriched mixed culture
Source: Front Microbiol. 2024 Aug 29;15:1438758. doi: 10.3389/fmicb.2024.1438758 (PMC11390636; doi:10.3389/fmicb.2024.1438758)
Supplement: Supplementary file 1 [file Data_Sheet_1.ZIP › 2. 16s rRNA sequencing.zip!/16s rRNA sequencing/Raw_data/assets/spgs/HN00197155.pdf]

Humanizing Genomics
macrogen

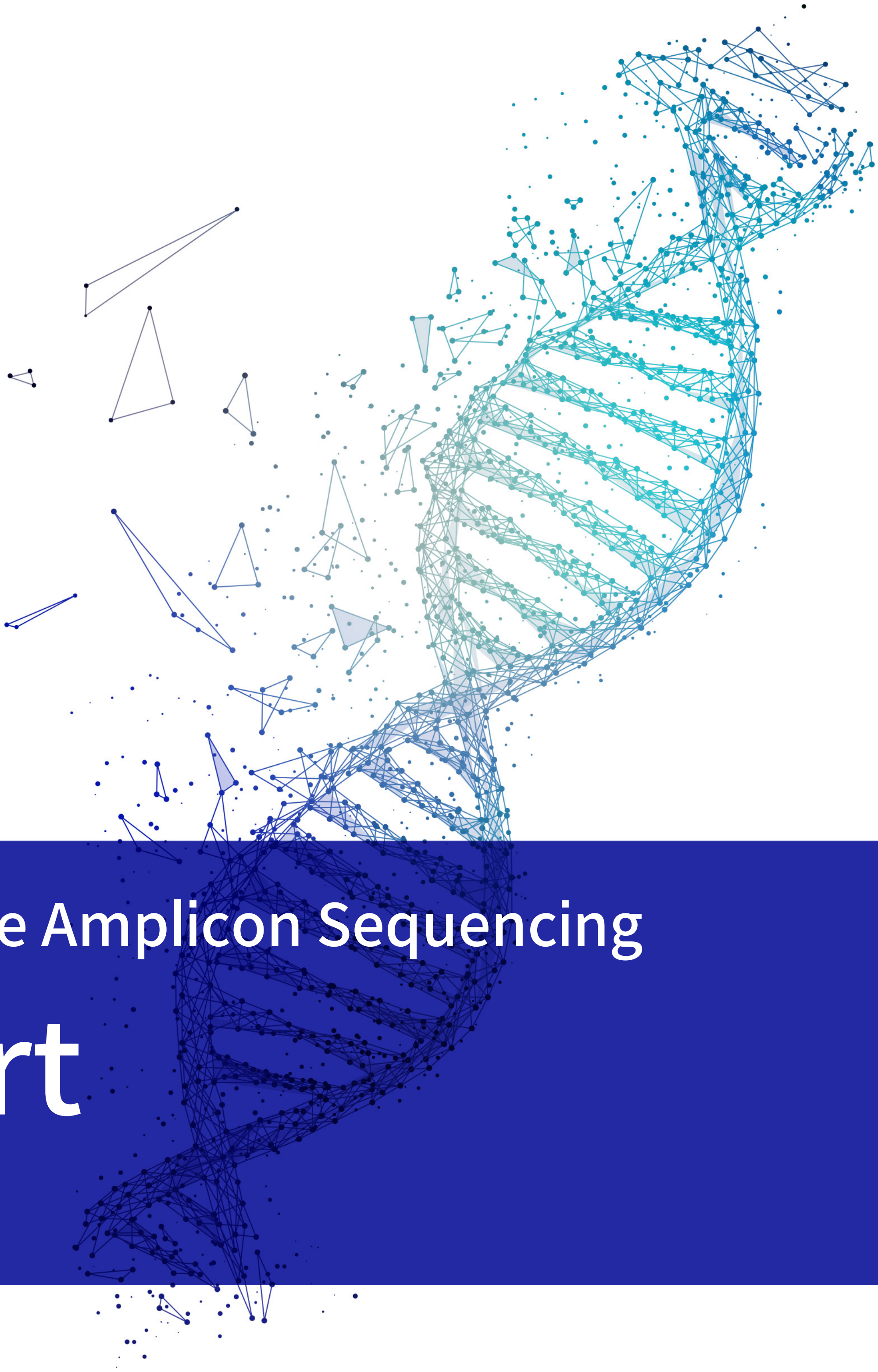

Metagenome Amplicon Sequencing

Report

2023.06

RAWDATA REPORT

Research use only

Table of Contents

Order Information 3

01 Workflow

Experimental Workflow 4

02 Raw Data Result

Raw Data Statistics 5
Total Bases 6
GC/AT Content 7
Q20/Q30 (%) 8

03 Deliverables

Download List 9

04 Appendix

FAQ 10
Result File Description 13

Order Information

Client Name	Macrogen Europe
Client Organization	Macrogen Europe
Order Number	HN00197155
Application	Metagenome Amplicon Sequencing
Type of Read	Paired-end
Read Length	301
Library Kit	Herculase II Fusion DNA Polymerase Nextera XT Index V2 Kit
Library Protocol	16S Metagenomic Sequencing Library Preparation Part # 15044223 Rev. B
Type of Sequencer	illumina system

Experimental Workflow

The samples are prepared according to NGS library preparation workflow, and sequenced using Illumina platform.
The workflow illustrated below shows the common ligation based method of library preparation. The process may differ based on the library preparation protocol followed.

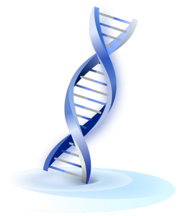

Sample Preparation

DNA/RNA is first extracted from the sample, and samples which meet quality control standards proceed to library construction.

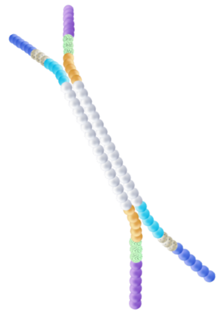

Ligate Adapters

The sequencing library is prepared by random fragmentation of the DNA or cDNA sample, followed by 5' and 3' adapter ligation. Alternatively, "tagmentation" combines the fragmentation and ligation reactions into a single step which greatly increases the efficiency of the library preparation process.

Final library Construction

Adapter-ligated fragments are then PCR amplified with a PCR primer solution which anneals to the ends of each adapters.
The library templates undergo quality control and quantification process.

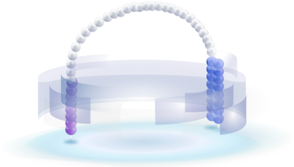

Cluster generation using bridge amplification

The library is loaded onto a flow cell where fragments are captured on a lawn of surface-bound oligos complementary to the library adapters.
Each fragment is then amplified into distinct clonal clusters through bridge amplification.
Once cluster generation is complete, the templates are ready for sequencing.

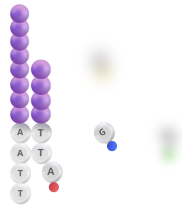

Sequencing by synthesis (SBS) technology

Illumina SBS technology utilizes a proprietary reversible terminator-based method that detects single bases as they are incorporated into DNA template strands. As all 4-reversible, terminator -bound dNTPs are present during each sequencing cycle, natural competition minimizes incorporation bias and greatly reduces raw error rates compared to other technologies.
The result is highly accurate base-by-base sequencing that virtually eliminates sequence-context-specific errors, even within repetitive sequence regions and homopolymers.

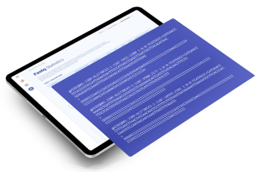

Generation of Raw data

The Illumina sequencer generates raw images utilizing sequencing control software for system control and base calling, through integrated primary analysis software called RTA (Real Time Analysis).
The BCL/cBCL (base call) binary files are converted into FASTQ files using bcl2fastq, which is an Illumina provided package. Adapters are not trimmed away from the reads.

Raw Data Statistics

• The total number of bases, reads, GC (%), Q20 (%), and Q30 (%) are calculated for the 2 samples.
For example, in COenrichment_V2 sample, 347,624 reads are produced, and total read bases are 104.6 Mbp.
The GC content (%) is 54.3% and Q30 is 76.2%.

* Raw Data

Sample ID	Total bases(bp)	Total reads	GC(%)	AT(%)	Q20(%)	Q30(%)
COenrichment_V2	104,634,824	347,624	54.3	45.7	87.0	76.2
COenrichment_V4	110,232,220	366,220	53.0	47.0	89.1	79.3

· Sample ID : Sample name.

· Total bases(bp) : Total number of bases sequenced.

· Total reads : Total number of reads. For illumina paired-end sequencing, this value refers to the sum of read1 and read2.

· GC(%) : Ratio of GC content.

· AT(%) : Ratio of AT content.

· Q20(%) : Ratio of bases that have phred quality score of over 20.

· Q30(%) : Ratio of bases that have phred quality score of over 30.

Total Bases Total number of samples : 2

* Raw Data

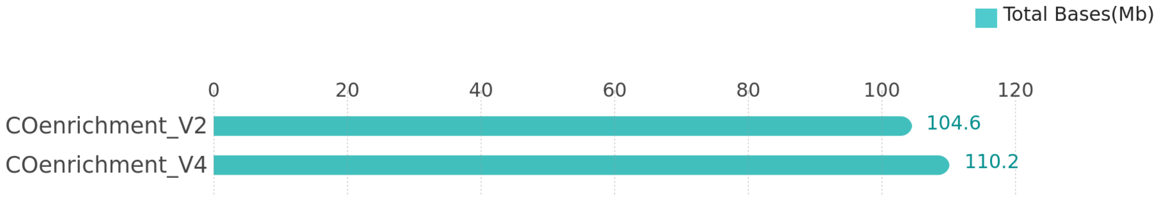

GC/AT Content
Total number of samples : 2

* Raw Data

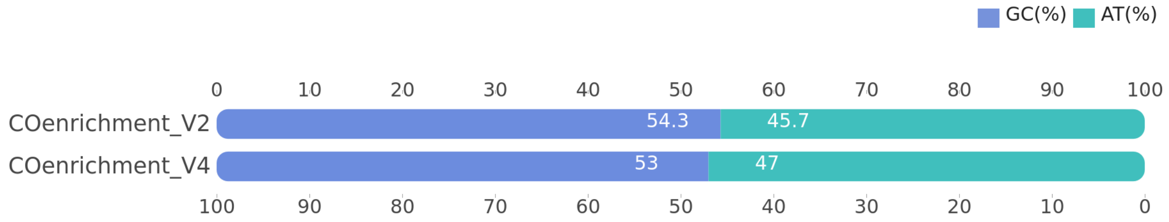

Q20/Q30 (%)

Total number of samples : 2

* Raw Data

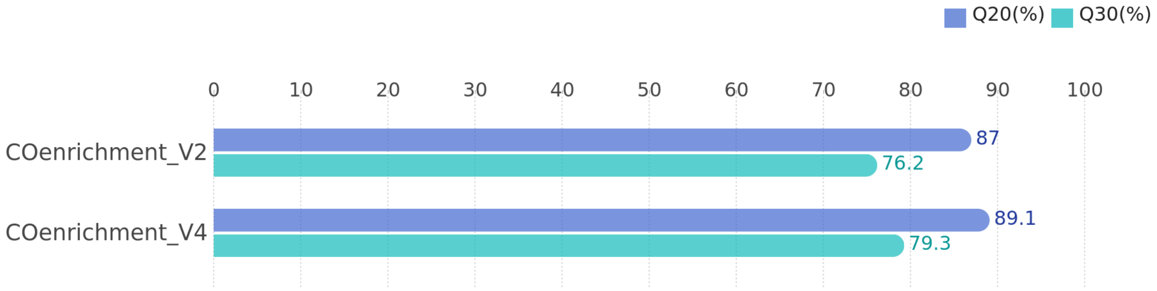

Download List

- The data can be downloaded from the links below. The download links are active for 2 weeks only, so please download your data within this period.
- Once you receive/download the data, please make sure to check the integrity of the files. Please note that the sequencing files will be deleted from our server 3 months after the analysis report is released; please contact us within 3 months if you encounter a problem with the data.

* Raw Data Download

File Name	File Size(byte)	md5sum
HN00197155.zip	105,125,182	f7542ea0a8226a4176d832f3490d770a

File Name	File Size(byte)	md5sum
COenrichment_V4_1.fastq.gz	21,312,826	65910345fb61cda9e86303e1d21d8cec
COenrichment_V4_2.fastq.gz	28,280,163	7317b9f307a256ee17d48c6b3b2e3485
COenrichment_V2_1.fastq.gz	24,962,579	863c2da75146be047c46f920b27361a4
COenrichment_V2_2.fastq.gz	30,568,872	73ac344ba5b1e458d955124bfeba719e

FAQ

Q **Why do I need to check the md5sum values, and how can I check it? (Windows system)**

A NGS data tend to have a large files size which makes them more likely to be corrupted during file transfer. So it's important that you check the md5sum of the files after receiving them to make sure what you received are what we gave.

Checking md5 hash in a Windows system

Windows does not provide a program for checking md5sum by default. An external program such as [QuickHash-Windows] can be used instead.

STEP 1 Download QuickHash-Windows from the website, and unzip the file.

STEP 2 Take a look at the UserManual.pdf file inside the zip file, and execute the .exe file.

Quickhash-GUI.exe	2,090,414	6,505,472
sqlite3-win32.dll	429,646	852,754
sqlite3-win64.dll	717,149	1,742,848
UserManual.pdf	512,697	576,987

STEP 3 Click on the "FileS" tab, and select [MD5] as the Algorithm.

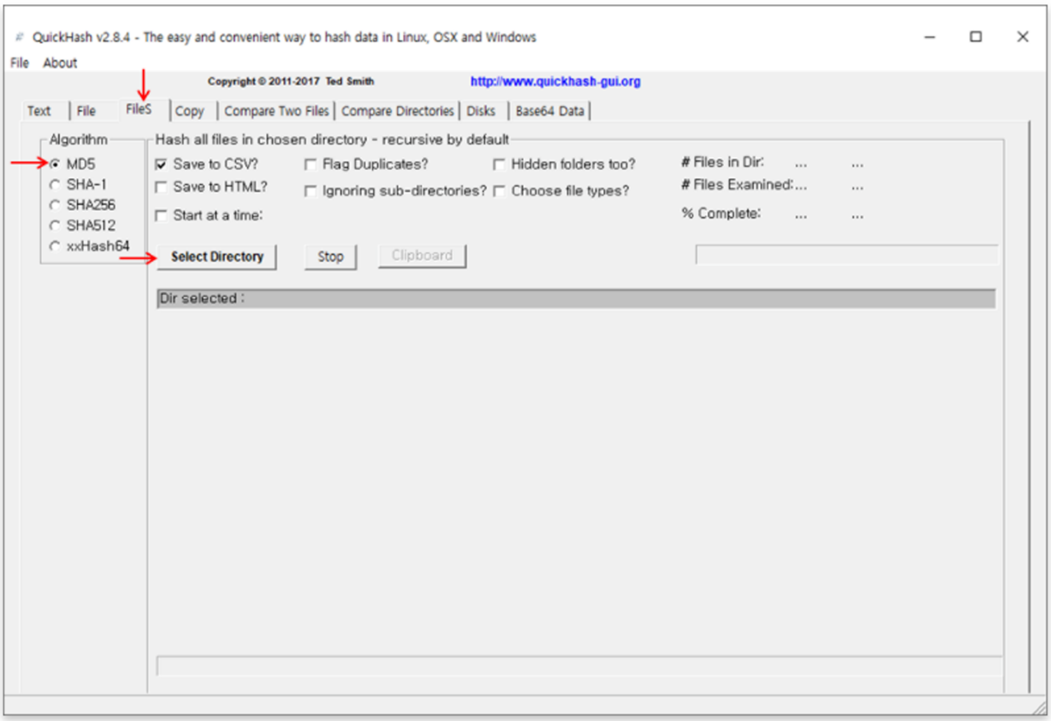

STEP 4 Click "Select Directory" and choose the directory where the files to be checked are located in.
The output can be saved as a csv or txt file.
The process may take some time depending on the performance of the system being used.

STEP 5 Compare the newly calculated md5 value with the md5 value provided to you through the Analysis Report.

FAQ

Q **Why do I need to check the md5sum values, and how can I check it? (Linux system)**

A NGS data tend to have a large files size which makes them more likely to be corrupted during file transfer. So it's important that you check the md5sum of the files after receiving them to make sure what you received are what we gave.

Checking md5 hash in a Linux system

Linux systems have an internal md5sum program under /user/bin/md5sum.
md5sum has a "-c" option, which reads the MD5 sums from the input file and checks them simultaneously.

Usage: $ md5sum -c [input file name]

STEP 1 Macrogen provides a text file containing the md5sum of deliverables you'll be receiving, which you can use to validate the integrity of the files. You can download this file by clicking on the "md5sum List" button in the "Download List" page. The text file will have the following name and format depending on how you're receiving your data:

- Via download link : **<OrderNumber>_#samples_md5sum_DownloadLink.txt**

```
[user@host] cat HN00000000_1samples_md5sum_DownloadLink.txt
File    Size    md5sum  Download_link
test_1.fastq.gz 3118212249      07a66a1d7d7fde2ee71b02a2caf21aba        https://data.macrogen.com/~macro3/HiSeq02//20210322/HN00000000/test_1.fastq.gz
test_2.fastq.gz 3305438294      3b4ff911e5d238a3c4763ee7967cb29a        https://data.macrogen.com/~macro3/HiSeq02//20210322/HN00000000/test_2.fastq.gz
[user@host]
```

- Via HDD : **<OrderNumber>_#samples_md5sum.txt**

```
[user@host] cat HN00000000_1samples_md5sum.txt
File    Size    md5sum
test_1.fastq.gz 3118212249      07a66a1d7d7fde2ee71b02a2caf21aba
test_2.fastq.gz 3305438294      3b4ff911e5d238a3c4763ee7967cb29a
[user@host]
```

- You can also find **"md5sum.txt"** located inside the HDD delivered to you.

```
[user@host]$ cat md5sum.txt
07a66a1d7d7fde2ee71b02a2caf21aba  RawData/test_1.fastq.gz
3b4ff911e5d238a3c4763ee7967cb29a  RawData/test_2.fastq.gz
[user@host]$
```

STEP 2 Use "md5sum -c" to validate the integrity of the file you've received. The input file for md5sum -c has to be delimited by two spaces with the md5sum column appearing before the file name, just like the sample image of "md5sum.txt" file shown above. As you can see, the two other files above are not formatted this way and need to altered to be used as input for md5sum -c. You can manually exclude the header and cut out "File" and "md5sum" column from the files, or simply run the following command:

$ awk '{print $3 " " $1}' <md5sum_file> | grep -v File

STEP 3 **"md5sum -c"** reads the input containing the md5 value of a file, and checks whether the md5 value of that file matches what's written inside the input file. This action outputs **"OK"** if the md5 value matches, and **"FAILED"** if otherwise. Check if the command outputs **"OK"** for all the files. (Refer to image below)

```
user@host
[user@host] awk '{print $3 "  " $1}' HN00000000_1samples_md5sum_DownloadLink.txt | grep -v File > md5sum.txt
[user@host] cat md5sum.txt
07a66a1d7d7fde2ee71b02a2caf21aba  test_1.fastq.gz
3b4ff911e5d238a3c4763ee7967cb29a  test_2.fastq.gz
[user@host]
[user@host] md5sum -c md5sum.txt
test_1.fastq.gz: OK
test_2.fastq.gz: OK
[user@host]
```

FAQ

Q I want to see the data produced by Macrogen. How can I open the files?

A NGS data tend to have large file sizes, and are not user-friendly to work with in a Windows environment.
We recommend that you use Linux system for smoother operation.

Q Where can I find information for Illumina adapter sequences?

A Information on Illumina adapters can be found in this support document:
Adapter Sequences Intro

Result File Description

Deliverables List

File Type	File Name	Description
**FASTQ**	[Sample name]_[read1].fastq.gz	Raw read1 sequence data
	[Sample name]_[read2].fastq.gz	Raw read2 sequence data
**md5sum**	[Order#]_[#samples]_md5sum[_DownloadLink].txt	You can download this file by clicking on the "md5sum List" button found on the "Download List" page. The file is slightly different in terms content, depending on how you're receiving your data. If you're receiving via download link, the file contains the following information : File name, File size, md5sum, FTP link. Otherwise, if your receiving your data via HDD the file only contains : File name, File size, and md5sum.<br><br>MD5 is a string of 32 hexadecimal values, which represents a 'fingerprint' of a file. By comparing the supplied MD5 value to the actual value computed by the MD5sums utility, you can make sure that the file that you downloaded off of the internet has not been tampered with or modified from the original file stored in our server.

FASTQ Format

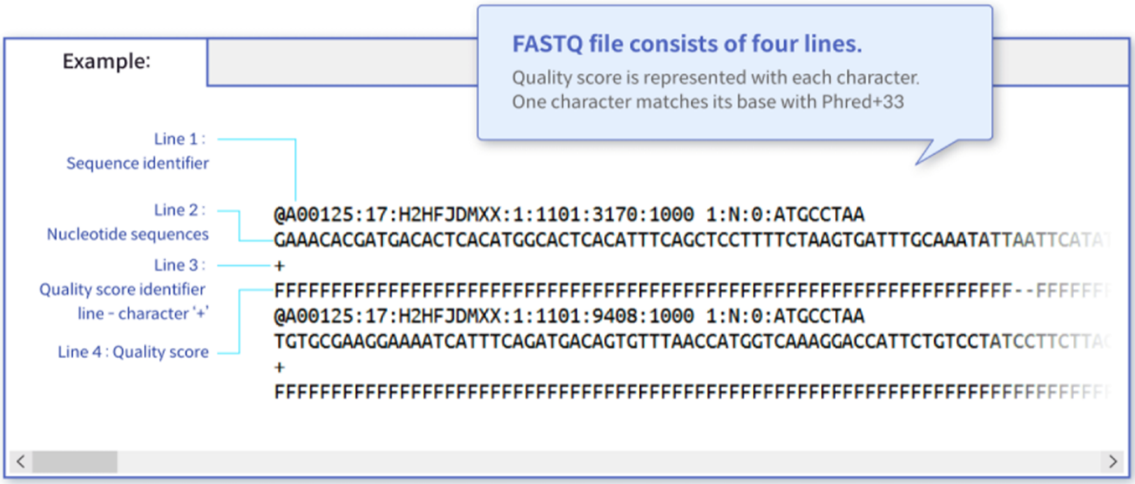

Phred Quality Score

Phred quality score numerically expresses the accuracy of each nucleotide. Higher Q number signifies higher accuracy.
For example, if Phred assigns a quality score of 30 to a base, the chances of having base call error are 1 in 1000.
Phred Quality Score Q is calculated with $-10\log_{10}(P)$, where *p* is probability of erroneous base call.

Quality of phred score	Probability of incorrect base call	Base call accuracy
10	1 in 10	90%
20	1 in 100	99%
30	1 in 1000	99.9%
40	1 in 10000	99.99%

Research use only

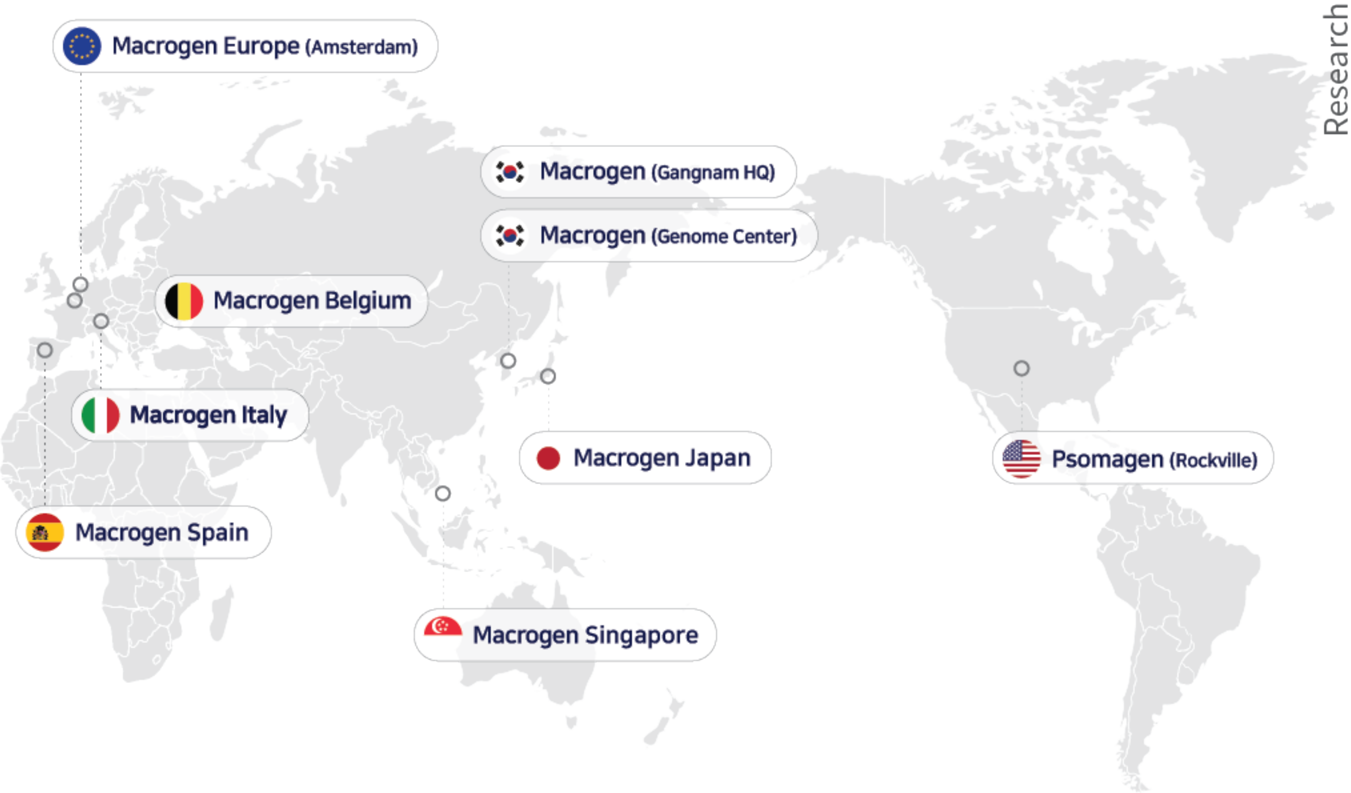

HEADQUARTER

Macrogen Gangnam HQ
Business & Support Center
Macrogen Bldg, 238, Teheran-ro,
Gangnam-gu, Seoul, Republic of Korea
Tel: +82-2-2180-7000
Web: www.macrogen.com
LIMS: dna.macrogen.com

Macrogen Genome Center
Laboratory & IT Center
[08511] 1001, 10F, 254, Beotkkot-ro,
Geumcheon-gu, Seoul, Republic of Korea
(Gasan-dong, World Meridian 1)
Tel: +82-2-2180-7000
Email1: ngs@macrogen.com(Overseas)
Email2: ngskr@macrogen.com
(Republic of Korea)
Web: www.macrogen.com
LIMS: dna.macrogen.com

SUBSIDIARY

Macrogen Europe
Laboratory,
Business & Support Center
Meibergdreef 57, 1105 BA, Amsterdam,
the Netherlands
Tel: +31-20-333-7563
Email: ngs@macrogen.eu

Psomagen (Macrogen USA)
Laboratory,
Business & Support Center
1330 Piccard Drive, Suite 103, Rockville,
MD 20850, United States
Tel: +1-301-251-1007
Email: inquiry@psomagen.com

Macrogen Singapore
Laboratory,
Business & Support Center
3 Biopolis Drive #05-18, Synapse,
Singapore 138623
Tel: +65-6339-0927
Email: info-sg@macrogen.com

Macrogen Japan
Laboratory,
Business & Support Center
16F Time24 Building, 2-4-32 Aomi,
Koto-ku, Tokyo 135-0064 JAPAN
Tel: +81-3-5962-1124
Email: ngs@macrogen-japan.co.jp

BRANCH

Macrogen Spain
Laboratory,
Business & Support Center
Av. Sur del Aeropuerto de Barajas,
28. Office B-2, 28042 Madrid, Spain
Tel: +34-911-138-378
Email: info-spain@macrogen.com

Macrogen Belgium
Laboratory,
Business & Support Center
Oxfordlaan 70, 6229 EV Maastricht,
Netherlands
Tel: +31-20-333-7563
Email: info.be@macrogen.eu

Macrogen Italy
Laboratory,
Business & Support Center
Viale Ortles, 22/4, 20139 Milano,
MI, Italy
Tel: +39-02-5666-0274
Email: italy@macrogen-europe.com

Humanizing Genomics
macrogen
