# Supplementary material for: Low electric current in a bioelectrochemical system facilitates ethanol production from CO using CO-enriched mixed culture
Source: Front Microbiol. 2024 Aug 29;15:1438758. doi: 10.3389/fmicb.2024.1438758 (PMC11390636; doi:10.3389/fmicb.2024.1438758)
Supplement: Supplementary file 1 [file Data_Sheet_1.ZIP › 2. 16s rRNA sequencing.zip!/16s rRNA sequencing/Raw_data/assets/spgs/xFAQ.html]

MACROGEN NGS Report


Order No.

- Workflow
  - Experimental Workflow
- Raw Data Result
  - Raw Data Statistics
  - Total Bases
  - GC/AT Content
  - Q20/Q30 (%)
  - Quality by Cycle
- Deliverables
  - Download List
- Appendix
  - FAQ
  - Result File Description


Research Use Only

Home

Appendix


- Workflow
- Raw Data Result
- Deliverables
- Appendix

FAQ


- FAQ
- Result File Description

FAQ

Why do I need to check the md5sum values, and how can I check it? (Windows system)

NGS data tend to have a large files size which makes them more likely to be corrupted during file transfer.  
So it's important that you check the md5sum of the files after receiving them to make sure what you  
received are what we gave.

Checking md5 hash in a Windows system

Windows does not provide a program for checking md5sum by default.
An external program such as QuickHash-Windows can be used instead.

STEP 1

Download QuickHash-Windows from the website, and unzip the file.

STEP 2

Take a look at the UserManual.pdf file inside the zip file, and execute the .exe file.

STEP 3

Click on the "FileS" tab, and select [MD5] as the Algorithm.

STEP 4

Click "Select Directory" and choose the directory where the files to be checked are located in.  
The output can be saved as a csv or txt file.  
The process may take some time depending on the performance of the system being used.

STEP 5

Compare the newly calculated md5 value with the md5 value provided to you through   
the Analysis Report.

Why do I need to check the md5sum values, and how can I check it? (Linux system)

NGS data tend to have a large files size which makes them more likely to be corrupted during file transfer.  
So it's important that you check the md5sum of the files after receiving them to make sure what you  
received are what we gave.

Checking md5 hash in a Linux system

Linux systems have an internal md5sum program under /user/bin/md5sum.  
md5sum has a "-c" option, which reads the MD5 sums from the input file and checks them simultaneously.

Usage: $ md5sum -c [input file name]

STEP 1

Macrogen provides a text file containing the md5sum of deliverables you'll be receiving, which you can use to validate the integrity of the files.
You can download this file by clicking on the "md5sum List" button in the "Download List" page. The text file will have the following name and format
depending on how you’re receiving your data:

○  Via download link : <OrderNumber>\_#samples\_md5sum\_DownloadLink.txt

○  Via HDD : <OrderNumber>\_#samples\_md5sum.txt

○  You can also find "md5sum.txt" located inside the HDD delivered to you.

STEP 2

Use "md5sum -c" to validate the integrity of the file you've received. The input file for md5sum -c has to be delimited by two spaces with the md5sum column appearing before the file name, just like the sample image of "md5sum.txt" file shown above. As you can see, the two other files above are not formatted this way and need to altered to be used as input for md5sum -c. You can manually exclude the header and cut out "File" and "md5sum" column from the files, or simply run the following command:
  
  

$ awk '{print $3 " " $1}' <md5sum\_file> | grep -v File

STEP 3

"md5sum -c" reads the input containing the md5 value of a file, and checks whether the md5 value of that file matches what’s written inside the input file. This action outputs "OK" if the md5 value
matches, and "FAILED" if otherwise. Check if the command outputs "OK" for all the files. (Refer to image below)

I want to see the data produced by Macrogen. How can I open the files?

NGS data tend to have large file sizes, and are not user-friendly to work with in a Windows environment.  
We recommend that you use Linux system for smoother operation.

Where can I find information for Illumina adapter sequences?

Information on Illumina adapters can be found in this support document:  
Adapter Sequences Intro

Order Information

|  |  |
| --- | --- |
|  |  |
|  |  |
|  | Order Number |

The data can be downloaded

from the link below.


The download link is active for 2 weeks only, 
so please download your data within this period.
Once you download the data, please make sure to check the integrity
of the files. The sequencing files will be deleted from our server
3 months after the analysis report is released;
please contact us within 3 months if you encounter a problem with the data.


Download Data

Company location

Macrogen Gangnam HQ

Business & Support Center

Macrogen Bldg, 238, Teheran-ro,  
Gangnam-gu, Seoul, Republic of Korea  
Tel: +82-2-2180-7000  
Web : https://www.macrogen.com  
LIMS : http://dna.macrogen.com

Macrogen Genome Center

Laboratory, IT and Business

[08511] 1001, 10F, 254, Beotkkot-ro,  
Geumcheon-gu, Seoul, Republic of Korea  
(Gasan-dong, World Meridian 1)  
Tel: +82-2-2180-7000  
Email1: ngs@macrogen.com(Overseas)  
Email2: ngskr@macrogen.com  
(Republic of Korea)  
Web : https://www.macrogen.com  
LIMS : http://dna.macrogen.com

Psomagen
(Macrogen USA)

Laboratory, Business & Support Center

1330 Piccard Drive, Suite 103, Rockville,  
MD 20850, United States  
T. +1-301-251-1007  
Email : inquiry@psomagen.com  
Web : https://psomagen.com

Macrogen Japan

Laboratory, Business & Support Center

16F Time24 Building, 2-4-32 Aomi,  
Koto-ku, Tokyo 135-0064 JAPAN  
T. +81-3-5962-1124  
E. ngs@macrogen-japan.co.jp

Macrogen Singapore

Laboratory, Business & Support Center

3 Biopolis Drive #05-18, Synapse,  
Singapore 138623  
T. +65-6339-0927  
Email : info-sg@macrogen.com

Macrogen Europe

Laboratory, Business & Support Center

Meibergdreef 57, 1105 BA, Amsterdam,  
the Netherlands  
T. +31-20-333-7563  
Email : ngs@macrogen.eu

Macrogen Spain

Laboratory, Business & Support Center

Av. Sur del Aeropuerto de Barajas,  
28, Office B-2, 28042 Madrid, Spain  
T. +34-911-138-378  
Email : info-spain@macrogen.com

Macrogen Belgium

Laboratory, Business & Support Center

Oxfordlaan 70, 6229 EV Maastricht,  
Netherlands  
Tel: +31-20-333-7563  
Email : info.be@macrogen.eu

Macrogen Italy

Viale Ortles, 22/4, 20139 Milano,   
MI, Italy  
Tel: +39-02-5666-0274  
Email : italy@macrogen-europe.com

TOP

Copyright © 
Research Use Only.
