# Supplementary material for: Low electric current in a bioelectrochemical system facilitates ethanol production from CO using CO-enriched mixed culture
Source: Front Microbiol. 2024 Aug 29;15:1438758. doi: 10.3389/fmicb.2024.1438758 (PMC11390636; doi:10.3389/fmicb.2024.1438758)
Supplement: Supplementary file 1 [file Data_Sheet_1.ZIP › 2. 16s rRNA sequencing.zip!/16s rRNA sequencing/Raw_data/assets/spgs/wExprmtWorkFlow.html]

MACROGEN NGS Report


Order No.

- Workflow
  - Experimental Workflow
- Raw Data Result
  - Raw Data Statistics
  - Total Bases
  - GC/AT Content
  - Q20/Q30 (%)
  - Quality by Cycle
- Deliverables
  - Download List
- Appendix
  - FAQ
  - Result File Description


Research Use Only

Home

Workflow


- Workflow
- Raw Data Result
- Deliverables
- Appendix

Experimental Workflow


- Experimental Workflow

Experimental Workflow

The samples are prepared according to NGS library preparation workflow, and sequenced using Illumina platform.  
The workflow illustrated below shows the common ligation based method of library preparation. The process may differ based  
on the library preparation protocol followed.

Overview

Library Preparation

Cluster Generation

Sequencing

Analysis

Library Preparation

Sample Preparation

DNA/RNA is first extracted from the sample,  
and samples which meet quality control standards  
proceed to library construction.

Ligate Adapters

The sequencing library is prepared by random fragmentation of the DNA or cDNA sample,  
followed by 5' and 3' adapter ligation. Alternatively, "tagmentation" combines the fragmentation  
and ligation reactions into a single step which greatly increases the efficiency of the library  
preparation process.

Final library Construction

Adapter-ligated fragments are then PCR amplified with a PCR primer solution which anneals  
to the ends of each adapters.  
The library templates undergo quality control and quantification process.

Cluster Generation

Cluster generation using bridge amplification

The library is loaded onto a flow cell where fragments are captured on  
a lawn of surface-bound oligos complementary to the library adapters.  
Each fragment is then amplified into distinct clonal clusters through bridge  
amplification. Once cluster generation is complete, the templates are ready  
for sequencing.

Sequencing

Sequencing by synthesis (SBS) technology

Illumina SBS technology utilizes a proprietary reversible terminator-based method that detects single bases as they are incorporated  
into DNA template strands. As all 4-reversible, terminator-bound dNTPs are present during each sequencing cycle, natural competition  
minimizes incorporation bias and greatly reduces raw error rates compared to other technologies.  
The result is highly accurate base-by-base sequencing that virtually eliminates sequence-context-specific errors, even within repetitive  
sequence regions and homopolymers.

|  |  |  |
| --- | --- | --- |
| \* MiSeq™ and HiSeq™ Series | \* MiniSeq™, NextSeq™, and NovaSeq™ Series | \* iSeq 100 System |

Four, Two, and One-Channel Chemistry

Systems using four-channel chemistry uses a mixture of nucleotides labeled with four different fluorescent dyes.  
Similarly, two-channel chemistry makes use of two different fluorescent dyes, and one-channel chemistry uses only one dye.  
The images are processed by an image analysis software to determine nucleotide identity.

Generation of Raw data

Generation of Raw data

The Illumina sequencer generates raw images utilizing sequencing control software for system control and base calling, through integrated  
primary analysis software called RTA (Real Time Analysis).  
The BCL/cBCL (base call) binary files are converted into FASTQ files using bcl2fastq, which is an Illumina provided package.  
Adapters are not trimmed away from the reads.

Order Information

|  |  |
| --- | --- |
|  |  |
|  |  |
|  | Order Number |

The data can be downloaded

from the link below.


The download link is active for 2 weeks only, 
so please download your data within this period.
Once you download the data, please make sure to check the integrity
of the files. The sequencing files will be deleted from our server
3 months after the analysis report is released;
please contact us within 3 months if you encounter a problem with the data.


Download Data

Company location

Macrogen Gangnam HQ

Business & Support Center

Macrogen Bldg, 238, Teheran-ro,  
Gangnam-gu, Seoul, Republic of Korea  
Tel: +82-2-2180-7000  
Web : https://www.macrogen.com  
LIMS : http://dna.macrogen.com

Macrogen Genome Center

Laboratory, IT and Business

[08511] 1001, 10F, 254, Beotkkot-ro,  
Geumcheon-gu, Seoul, Republic of Korea  
(Gasan-dong, World Meridian 1)  
Tel: +82-2-2180-7000  
Email1: ngs@macrogen.com(Overseas)  
Email2: ngskr@macrogen.com  
(Republic of Korea)  
Web : https://www.macrogen.com  
LIMS : http://dna.macrogen.com

Psomagen
(Macrogen USA)

Laboratory, Business & Support Center

1330 Piccard Drive, Suite 103, Rockville,  
MD 20850, United States  
T. +1-301-251-1007  
Email : inquiry@psomagen.com  
Web : https://psomagen.com

Macrogen Japan

Laboratory, Business & Support Center

16F Time24 Building, 2-4-32 Aomi,  
Koto-ku, Tokyo 135-0064 JAPAN  
T. +81-3-5962-1124  
E. ngs@macrogen-japan.co.jp

Macrogen Singapore

Laboratory, Business & Support Center

3 Biopolis Drive #05-18, Synapse,  
Singapore 138623  
T. +65-6339-0927  
Email : info-sg@macrogen.com

Macrogen Europe

Laboratory, Business & Support Center

Meibergdreef 57, 1105 BA, Amsterdam,  
the Netherlands  
T. +31-20-333-7563  
Email : ngs@macrogen.eu

Macrogen Spain

Laboratory, Business & Support Center

Av. Sur del Aeropuerto de Barajas,  
28, Office B-2, 28042 Madrid, Spain  
T. +34-911-138-378  
Email : info-spain@macrogen.com

Macrogen Belgium

Laboratory, Business & Support Center

Oxfordlaan 70, 6229 EV Maastricht,  
Netherlands  
Tel: +31-20-333-7563  
Email : info.be@macrogen.eu

Macrogen Italy

Viale Ortles, 22/4, 20139 Milano,   
MI, Italy  
Tel: +39-02-5666-0274  
Email : italy@macrogen-europe.com

TOP

Copyright © 
Research Use Only.
