# Supplementary material for: Low electric current in a bioelectrochemical system facilitates ethanol production from CO using CO-enriched mixed culture
Source: Front Microbiol. 2024 Aug 29;15:1438758. doi: 10.3389/fmicb.2024.1438758 (PMC11390636; doi:10.3389/fmicb.2024.1438758)
Supplement: Supplementary file 1 [file Data_Sheet_1.ZIP › 2. 16s rRNA sequencing.zip!/16s rRNA sequencing/HN00197155_OTU_2/OTU_Results/Diversity_Index/adiv.html]

Community richness & diversity


# NGS Analysis Report - OTU

## Community richness & diversity

Community richness & diversity

| OTUs | | | | | |  |
| --- | --- | --- | --- | --- | --- | --- |
|  | | | | | |  |
| Community Diversity | | | | | |  |
|  | | | | | |  |
| SampleName | OTUs | Chao1 | Shannon | Gini-Simpson | Good's Coverage |  |
| COenrichment.V4 | 21.0 | 21.0 | 0.403746353574 | 0.110052157105 | 1.0 |  |
| \* OTUs : Operational Taxonomic Unit is an operational definition of a species or group of species often used when only DNA sequence data is available  \* Chao1 : returns the Chao1 richness estimate for an OTU definition  \* Shannon : The Shannon index takes into account the number and evenness of species.  \* Gini-Simpson : The Gini-Simpson index represents the probability that two randomly selected individuals in the habitat will belong to the same species.  \* Good's Coverage : Coverage is calculated as *C=1-(s/n)*,        where *s* is the number of unique OTUs and *n* is the number of individuals in the sample.        This index gives a relative measure of how well the sample represents the larger environment. | | | | | | |
