# Supplementary material for: Low electric current in a bioelectrochemical system facilitates ethanol production from CO using CO-enriched mixed culture
Source: Front Microbiol. 2024 Aug 29;15:1438758. doi: 10.3389/fmicb.2024.1438758 (PMC11390636; doi:10.3389/fmicb.2024.1438758)
Supplement: Supplementary file 1 [file Data_Sheet_1.ZIP › 2. 16s rRNA sequencing.zip!/16s rRNA sequencing/HN00197155_OTU_2/OTU_Results/Alpha_rarefaction/alpha_rarefaction_plots/rarefaction_plots.html]

Rarefaction Curve


# NGS Analysis Report - OTU

## Rarefaction Curve Graph

- Alpha rarefaction graph shows whether the number of reads used in analysis was sufficient in identifying species/OTU.  
  If the curve becomes flatter to the right, it indicates that a reasonable number of reads have been used in the analysis,thus additional sequencing is not necessary.  
  In contrast, if the graph does not plateau, the additional reads is likely to discover more OTUs for the sample (x-axis: read number; y-axis: number of OTUS).  
  Below each plot is a table displaying average values for each measure of alpha diversity for each group of samples the specified category.  
    
  \* Metric : PD\_whole\_tree, chao1, observed\_species(OTUs)  
  \* Category : SampleID, Group, etc
  


|  |  |  |  |
| --- | --- | --- | --- |
| **Select a Metric:** | PD\_whole\_tree chao1 observed\_species | **Select a Category:** | SampleID |

  

**Show Categories:
 
All
None
Invert**

**Legend**

|  |  |  |  |
| --- | --- | --- | --- |
| ▶ |  | ■ | **COenrichment.V4** |
| ∟ |  | ◆ | **COenrichment.V4** |
| ▶ |  | ■ | **COenrichment.V4** |
| ∟ |  | ◆ | **COenrichment.V4** |
| ▶ |  | ■ | **COenrichment.V4** |
| ∟ |  | ◆ | **COenrichment.V4** |

**If the lines for some categories do not extend all the way to the right end of the x-axis, that means that at least one of the samples in that category does not have that many sequences.**
