# Supplementary material for: Low electric current in a bioelectrochemical system facilitates ethanol production from CO using CO-enriched mixed culture
Source: Front Microbiol. 2024 Aug 29;15:1438758. doi: 10.3389/fmicb.2024.1438758 (PMC11390636; doi:10.3389/fmicb.2024.1438758)
Supplement: Supplementary file 1 [file Data_Sheet_1.ZIP › 2. 16s rRNA sequencing.zip!/16s rRNA sequencing/HN00197155_OTU_1/OTU_Results/Taxonomy_assignment_blast_NCBI_16S/taxa_summary_plots/bar_charts.html]

Taxa Summaries


# NGS Analysis Report - OTU

## Taxonomic assignment

- The following chart shows the taxonomic composition for each sample from phylum to genus levels (x-axis: sample name;y-axis: OTU proportions).  
  You can mouse over the plot to see which taxa are contributing to the percentage shown

- Phylum
- Class
- Order
- Family
- Genus
- Species

|  |  |
| --- | --- |
| Phylum Level | |
| View Figure (.pdf)  View Legend (.pdf) |  |
|  |  |


View Table (.txt)

|  |  |  |  |
| --- | --- | --- | --- |
|  | | Total | COenrichment.V2 |
| Legend | Taxonomy | % | % |
|  | Bacteria;\_\_Bacillota | 99.6% | 99.6% |
|  | \_\_;\_\_ | 0.4% | 0.4% |

|  |  |
| --- | --- |
| Class Level | |
| View Figure (.pdf)  View Legend (.pdf) |  |
|  |  |


View Table (.txt)

|  |  |  |  |
| --- | --- | --- | --- |
|  | | Total | COenrichment.V2 |
| Legend | Taxonomy | % | % |
|  | Bacteria;\_\_Bacillota;\_\_Bacilli | 9.3% | 9.3% |
|  | Bacteria;\_\_Bacillota;\_\_Clostridia | 90.3% | 90.3% |
|  | \_\_;\_\_;\_\_ | 0.4% | 0.4% |

|  |  |
| --- | --- |
| Order Level | |
| View Figure (.pdf)  View Legend (.pdf) |  |
|  |  |


View Table (.txt)

|  |  |  |  |
| --- | --- | --- | --- |
|  | | Total | COenrichment.V2 |
| Legend | Taxonomy | % | % |
|  | Bacteria;\_\_Bacillota;\_\_Bacilli;\_\_Lactobacillales | 9.3% | 9.3% |
|  | Bacteria;\_\_Bacillota;\_\_Clostridia;\_\_Eubacteriales | 90.3% | 90.3% |
|  | \_\_;\_\_;\_\_;\_\_ | 0.4% | 0.4% |

|  |  |
| --- | --- |
| Family Level | |
| View Figure (.pdf)  View Legend (.pdf) |  |
|  |  |


View Table (.txt)

|  |  |  |  |
| --- | --- | --- | --- |
|  | | Total | COenrichment.V2 |
| Legend | Taxonomy | % | % |
|  | Bacteria;\_\_Bacillota;\_\_Bacilli;\_\_Lactobacillales;\_\_Enterococcaceae | 1.8% | 1.8% |
|  | Bacteria;\_\_Bacillota;\_\_Bacilli;\_\_Lactobacillales;\_\_Lactobacillaceae | 7.5% | 7.5% |
|  | Bacteria;\_\_Bacillota;\_\_Clostridia;\_\_Eubacteriales;\_\_Clostridiaceae | 89.2% | 89.2% |
|  | Bacteria;\_\_Bacillota;\_\_Clostridia;\_\_Eubacteriales;\_\_Oscillospiraceae | 1.2% | 1.2% |
|  | \_\_;\_\_;\_\_;\_\_;\_\_ | 0.4% | 0.4% |

|  |  |
| --- | --- |
| Genus Level | |
| View Figure (.pdf)  View Legend (.pdf) |  |
|  |  |


View Table (.txt)

|  |  |  |  |
| --- | --- | --- | --- |
|  | | Total | COenrichment.V2 |
| Legend | Taxonomy | % | % |
|  | Bacteria;\_\_Bacillota;\_\_Bacilli;\_\_Lactobacillales;\_\_Enterococcaceae;\_\_Enterococcus | 1.8% | 1.8% |
|  | Bacteria;\_\_Bacillota;\_\_Bacilli;\_\_Lactobacillales;\_\_Lactobacillaceae;\_\_Lacticaseibacillus | 7.5% | 7.5% |
|  | Bacteria;\_\_Bacillota;\_\_Clostridia;\_\_Eubacteriales;\_\_Clostridiaceae;\_\_Clostridium | 89.2% | 89.2% |
|  | Bacteria;\_\_Bacillota;\_\_Clostridia;\_\_Eubacteriales;\_\_Oscillospiraceae;\_\_Caproicibacter | 1.1% | 1.1% |
|  | Bacteria;\_\_Bacillota;\_\_Clostridia;\_\_Eubacteriales;\_\_Oscillospiraceae;\_\_Ethanoligenens | 0.0% | 0.0% |
|  | Bacteria;\_\_Bacillota;\_\_Clostridia;\_\_Eubacteriales;\_\_Oscillospiraceae;\_\_Massiliimalia | 0.0% | 0.0% |
|  | \_\_;\_\_;\_\_;\_\_;\_\_;\_\_ | 0.4% | 0.4% |

|  |  |
| --- | --- |
| Species Level | |
| View Figure (.pdf)  View Legend (.pdf) |  |
|  |  |


View Table (.txt)

|  |  |  |  |
| --- | --- | --- | --- |
|  | | Total | COenrichment.V2 |
| Legend | Taxonomy | % | % |
|  | Bacteria;\_\_Bacillota;\_\_Bacilli;\_\_Lactobacillales;\_\_Enterococcaceae;\_\_Enterococcus;\_\_Enterococcus hirae | 1.8% | 1.8% |
|  | Bacteria;\_\_Bacillota;\_\_Bacilli;\_\_Lactobacillales;\_\_Lactobacillaceae;\_\_Lacticaseibacillus;\_\_Lacticaseibacillus paracasei | 7.5% | 7.5% |
|  | Bacteria;\_\_Bacillota;\_\_Clostridia;\_\_Eubacteriales;\_\_Clostridiaceae;\_\_Clostridium;\_\_Clostridium autoethanogenum | 89.1% | 89.1% |
|  | Bacteria;\_\_Bacillota;\_\_Clostridia;\_\_Eubacteriales;\_\_Clostridiaceae;\_\_Clostridium;\_\_Clostridium neuense | 0.0% | 0.0% |
|  | Bacteria;\_\_Bacillota;\_\_Clostridia;\_\_Eubacteriales;\_\_Oscillospiraceae;\_\_Caproicibacter;\_\_Caproicibacter fermentans | 1.1% | 1.1% |
|  | Bacteria;\_\_Bacillota;\_\_Clostridia;\_\_Eubacteriales;\_\_Oscillospiraceae;\_\_Ethanoligenens;\_\_Ethanoligenens harbinense | 0.0% | 0.0% |
|  | Bacteria;\_\_Bacillota;\_\_Clostridia;\_\_Eubacteriales;\_\_Oscillospiraceae;\_\_Massiliimalia;\_\_Massiliimalia massiliensis | 0.0% | 0.0% |
|  | \_\_;\_\_;\_\_;\_\_;\_\_;\_\_;Other | 0.4% | 0.4% |
