# Supplementary material for: Low electric current in a bioelectrochemical system facilitates ethanol production from CO using CO-enriched mixed culture
Source: Front Microbiol. 2024 Aug 29;15:1438758. doi: 10.3389/fmicb.2024.1438758 (PMC11390636; doi:10.3389/fmicb.2024.1438758)
Supplement: Supplementary file 1 [file Data_Sheet_1.ZIP › 2. 16s rRNA sequencing.zip!/16s rRNA sequencing/HN00197155_OTU_2/OTU_Results/Taxonomy_assignment_blast_NCBI_16S/taxa_summary_plots/charts/5bCsz23zlE2BeIB6SCuH15zRyLF0Ek_legend.pdf]

Bacteria;__Bacillota;__Bacilli;__Lactobacillales;__Enterococcaceae
Bacteria;__Bacillota;__Bacilli;__Lactobacillales;__Lactobacillaceae
Bacteria;__Bacillota;__Clostridia;__Eubacteriales;__Clostridiaceae
Bacteria;__Bacillota;__Clostridia;__Eubacteriales;__Oscillospiraceae
Bacteria;__Bacillota;__Clostridia;__Thermoanaerobacterales;__Thermoanaerobacteraceae
